# Cholinergic Receptor Nicotinic Alpha 5 (CHRNA5) RNAi is associated with cell cycle inhibition, apoptosis, DNA damage response and drug sensitivity in breast cancer

**DOI:** 10.1371/journal.pone.0208982

**Published:** 2018-12-13

**Authors:** Sahika Cingir Koker, Ermira Jahja, Huma Shehwana, Ayse Gokce Keskus, Ozlen Konu

**Affiliations:** 1 Department of Molecular Biology and Genetics, Faculty of Science, Bilkent University, Ankara, Turkey; 2 Department of Multidisciplinary Studies, National University of Medical Sciences, Rawalpindi, Pakistan; 3 Interdisciplinary Neuroscience Program, Bilkent University, Ankara, Turkey; 4 UNAM-Institute of Materials Science and Nanotechnology, Ankara, Turkey; Wayne State University, UNITED STATES

## Abstract

Cholinergic Receptor Nicotinic Alpha 5 (CHRNA5) is an important susceptibility locus for nicotine addiction and lung cancer. Depletion of CHRNA5 has been associated with reduced cell viability, increased apoptosis and alterations in cellular motility in different cancers yet not in breast cancer. Herein we first showed the expression of CHRNA5 was variable and positively correlated with the fraction of total genomic alterations in breast cancer cell lines and tumors indicating its potential role in DNA damage response (DDR). Next, we demonstrated that silencing of CHRNA5 expression in MCF7 breast cancer cell line by RNAi affected expression of genes involved in cytoskeleton, TP53 signaling, DNA synthesis and repair, cell cycle, and apoptosis. The transcription profile of CHRNA5 depleted MCF7 cells showed a significant positive correlation with that of A549 lung cancer cell line while exhibiting a negative association with the CHRNA5 co-expression profile obtained from Cancer Cell Line Encylopedia (CCLE). Moreover, it exhibited high similarities with published MCF7 expression profiles obtained from exposure to TP53 inducer nutlin-3a and topoisomerase inhibitors. We then demonstrated that CHRNA5 siRNA treatment reduced cell viability and DNA synthesis indicating G1 arrest while it significantly increased apoptotic sub-G1 cell population. Accordingly, we observed lower levels of phosphorylated RB (Ser807/811) and an increased BAX/BCL2 ratio in RNAi treated MCF7 cells. We also showed that CHRNA5 RNAi transcriptome correlated negatively with DDR relevant gene expression profile in breast cancer gene expression datasets while the coexposure to topoisomerase inhibitors in the presence of CHRNA5 RNAi enhanced chemosensitivity potentially due to reduced DDR. CHRNA5 RNAi consistently lowered total CHEK1 mRNA and protein levels as well as phosphorylated CHEK1 (Ser345) in MCF7 cells. We also detected a significant positive correlation between the expression levels of CHRNA5 and CHEK1 in CCLE, TCGA and METABRIC breast cancer datasets. Our study suggests CHRNA5 RNAi is associated with cell cycle inhibition, apoptosis as well as reduced DDR and increased drug sensitivity in breast cancer yet future studies are warranted since dose- and cell line-specific differences exist in response to CHRNA5 depletion. Gene expression microarray data can be accessed from GEO database under the accession number GSE89333.

## Introduction

Nicotinic acetylcholine receptors (nAChRs), which have a pentameric structure, are ligand gated ion channels and can be activated by acetylcholine or nicotine [1]. Their activity as well as subunit composition are known to play significant roles in lung cancer progression [2] either by altering proliferation of lung cancer cells or rendering them resistant to apoptotic signals and hence chemotherapic agents [3, 4]. Chromosomal locus 15q25 is home for several nAChR genes, i.e., CHRNA5, CHRNA3, and CHRNB4 [5]. Among them CHRNA5 has been implicated in drug addiction as well as cancer development [6]. For example, the transcription of CHRNA5 can be modulated by alcohol in human embryonic cells [7] and in response to nicotine exposure in bronchial epithelial cell lines [8] and in lung cancer [9]. In addition, splicing factors, such as ESRP1 and ESRP2, contribute to the alternative splicing of CHRNA5 by repressing downstream 5' splice sites in exon 5 [10]. The resultant CHRNA5 isoforms are shown to be elevated in lung and gastric cancers [11, 12] also making CHRNA5 a potential cancer biomarker.

The present literature further suggests that CHRNA5 may act as an oncogene possibly through its potential role in DNA damage response (DDR) as well as alteration of known cancer signaling pathways such as JAK/STAT, PI3K/AKT and Ras/Raf/MEK/ERK [13]. A recent study shows that elevated levels of CHRNA5 are associated with increased DNA adduct formation in connection with the TP53 mutation status in non-small cell lung carcinoma (NSCLC) [1]. Increased CHRNA5 levels correlate with phosphorylated STAT3 levels in NSCLC implicating an important role for CHRNA5 in modulation of cellular signaling in cancer [9]. RNAi against CHRNA5 or STAT3 can lead to inhibition of cellular proliferation in NSCLC cell lines; and a mouse xenograft study shows that tumors derived from a CHRNA5-depleted small cell lung carcinoma cell line have lower size and weight in comparison to the controls [14]. Moreover depletion of CHRNA5 alters cell adhesion and activity of p63, an important molecule with established roles in cellular differentiation [15] in squamous cell lung carcinoma where increased CHRNA5 levels are detected especially in smokers and in poorly differentiated tumors [16].

Inherent or acquired resistance to drugs is an important effector in differential patient survival and prognosis. Therefore, sensitizing cancer cells to common anti-cancer drugs using siRNA molecules is a promising avenue in the development of cancer therapeutics [17]. CHRNA5 can have potential implications in drug sensitivity since a recent study in gastric cancer shows that depletion of CHRNA5 hinders the pro-proliferative role of nicotine and resensitizes cells to cisplatin [12]. Similarly, CHRNA5 mediates nicotine’s activity on intracellular calcium response and can modulate drug resistivity [18, 19]. However, molecular associations of this chemosensitivity remain to be elucidated.

Reduced DDR is one of the major requirements of increased drug sensitivity [20, 21]. DDR requires phosphorylation of both CHEK1 and H2AX by ATR for initiation of a primary response [22–24]. Activation of CHEK1 then can lead to cell cycle arrest, DNA repair or apoptosis. Furthermore, phopshorylation of CDC25A by CHEK1 leads to CDC25A degradation and hence inactivation of CDK2 resulting in G1/S arrest. Recent studies also show that CHEK1 is required for the unperturbed cell cycle and relief from DNA damage stress [25, 26]. Moreover, depletion of CHEK1 is known to sensitize cells to DNA damage inducing reagents [27, 28]. Accordingly, CHEK1 inhibitors applied with DNA damage inducing reagents or anti-mitotics exhibit additive effects. However, there is no study examining the association of CHRNA5 with CHEK1 or DDR in breast cancer.

Breast cancer is one of the main causes of mortality among women [29] and heterogenous in nature presenting with various molecular subtypes and differential prognosis [30]. TP53 status along with DDR pathways also have been shown to be valuable for diagnosis and prognosis of breast tumors [31, 32]. Breast cancer cell lines exhibit cholinergic signaling by responding to nicotine and express nicotinic acetylcholine receptors including CHRNA5, 7, and 9 [33, 34]. However, the expression pattern of CHRNA5 in breast cancer cell lines and its possible relationship with genomic alteration levels, as an indirect measurement of DDR [35], has not been studied. Moreover, the effects of CHRNA5 depletion by RNAi have yet to be tested on breast cancer cell proliferation, apoptosis, DDR and hence drug sensitivity.

Herein we showed the differential expression of CHRNA5 in breast cancer cell lines and tumors with respect to the fraction of genomic alterations and TP53 mutation status. To test the effects of CHRNA5 depletion we used a CHRNA5 RNAi model in MCF7 cells and performed microarray expression analysis showing coordinated transcriptomic changes in multiple pathways involved in tumor formation and progression. Functional analysis of CHRNA5 coexpression profile across CCLE breast cancer cell lines revealed a positive association especially with cell cycle regulation and DNA repair pathways. A significant correlation between the CHRNA5 RNAi expression profile in MCF7 cells and that in A549 lung cell line was also observed. Comparative transcriptomics with published *in vitro* and breast cancer patient datasets strongly pointed to the role of CHRNA5 RNAi in cell cycle regulation, and TP53 and DDR signaling. In the presence of CHRNA5 RNAi we observed significant reductions in cell viability, DNA synthesis and phosphorylated RB (Ser807/811) levels accompanied by reduced CCNE2 mRNA levels, indicative of cell cycle arrest. The increase in sub-G1 population by CHRNA5 RNAi exposure was associated with significantly higher BAX/BCL2 ratios. We next demonstrated that CHRNA5 RNAi reduced both the total CHEK1 and phosphorylated CHEK1 levels in the presence of camptothecin (CPT) and/or doxorubicin (DOXO) in MCF7 cells. However, MDA-MB-231 and BT-20 cells exhibited increased phosphorylated CHEK1 levels in response to CPT and DOXO while CHRNA5 RNAi resulted in a reduction in CHEK1 levels only in MDA-MB-231 cells. A strong positive correlation was observed also between mRNA expressions of CHRNA5 and CHEK1 across breast cancer cell lines and tumors. Overall, our findings suggest that CHRNA5 depletion is associated with anti-proliferation, cell cycle arrest, apoptosis as well as reduced DDR and increased drug sensitivity in breast cancer.

## Materials and methods

### Cell culture and RNAi treatment

MCF7, BT-20 and MDA-MB-231 breast cancer cell lines (ATCC) were used in this study. MCF7 cells (2 x 10^5^), seeded in 6-well plates were treated after 24h either with an siRNA against several CHRNA5 spliced variants (FlexiTube, SI03051111, Qiagen; siRNA-1) or the respective negative control at the same concentration (AllStars Negative Control siRNA, 1027280, Qiagen; siRNA-CN) using HiPerFect transfection reagent (6:1000; transfection reagent: total volume; 301704, Qiagen). Another control with no treatment was also used. Experiments were performed using two or more biological replicas. Treatments were performed for 72h and 120h when needed, and cell pellets were stored at -80 ºC until further use. In addition to siRNA-1, two other siRNA molecules (FlexiTube, SI03099453, siRNA-2; and FlexiTube, SI03096940, siRNA-3) were tested for validation. All siRNAs targeted nearby regions of CHRNA5 exon 5 (Gene accession: NM_000745), parts of which are alternatively spliced. Doses and exposure times were optimized as 10nM 72h for siRNA-1 and 50nM 72h for siRNA-2 and -3. An additional 48h transfection of siRNA-1 (for a total of 120h) was perfomed to study the prolonged downregulation of CHRNA5 in MCF7 cell line. Treatments were performed in duplicates. siRNA-1 and corresponding siRNA-CN exposures also were performed in BT-20 (10nM) and MDA-MB-231 (50nM) cells as described for MCF7 cells. RNAi experiments for DDR response were conducted with topoisomerase inhibitors, camptothecin (CPT) and doxorubicin (DOXO) in MCF7, BT-20 and MDA-MB-231 cell lines at concentrations determined by MTT assays which were indicated in the corresponding figure legends.

### RNA isolation, cDNA synthesis, RT-qPCR

RNA was isolated using RNeasy Mini kit (74104, Qiagen) and 1μg total RNA was reverse transcribed into cDNA (RevertAid first strand cDNA synthesis kit, K1622, Fermentas). RT-qPCR was performed with SYBR Green mix (04707516001, Roche) using LightCycler 480 (Roche). SDHA was used as a reference gene. Primer efficiencies were determined using serially diluted cDNA as either 1:10, 1:100, 1:1000, 1:10000, 1:5, 1:25, 1:125, 1:625 or 1:2, 1:4, 1:8, 1:16, 1:32, 1:64 folds (for genes with low basal expression level) (see for primers; S1 Table).

### Primers for CHRNA5 isoforms

Multiple CHRNA5 isoforms were amplified from MCF7 cells by RT-qPCR using the following primer set (Forward 5’-GGAGATACCCTGATGATGACTTAA-3’, reverse 5’-AAAAAGAAGCCCAGAAACAATTCC-3’) [10]. The full-length (*CHRNA_v2*) and shortest (*CHRNA_v3*) amplicons after being extracted from 1.5% agarose gels using Agarose Gel Extraction Kit (11696505001, Roche) were cloned into the pGEM-T Easy vector (A1360, Promega) according to the manufacturer’s instructions. Sequencing was performed using the SP6 primer and BLAST search identified the hit as the CHRNA5 mRNA NCBI (NM_000745.3). To amplify the CHRNA5 mRNA by RT-qPCR we used specific primers for *CHRNA5_v2* and *CHRNA5_v3* isoforms, another primer pair amplifying multiple isoforms of CHRNA5 (*CHRNA5_v1*) and two other isoform-specific primer pairs previously reported (*Iso2* and *Iso3*; S1 Table) [11].

### Transcriptome analysis of CHRNA5 siRNA treatment in MCF7 cells

The Affymetrix HG-U133 Plus 2 Platform was used for expression profiling of siRNA-1 (10nM) and corresponding siRNA-CN treated MCF7 cells (n = 2 per group) according to the manufacturer’s protocols. Data were obtained using GeneChip Operating Software (Affymetrix HG-U133 plus 2). Quality control was performed via *affyQCReport* package in R [36]. Cel files were *rma* normalized using *affy* package [37]. To remove multiple probesets for each gene, only jetset (Version: 3.1.2) best probesets [38] (20089 probesets) were subjected to functional annotation analysis with GSEA and differential expression analysis using *limma* package [39, 40]. Multiple test corrections were performed by Benjamini and Hochberg (BH) method [41] and genes having BH < 0.05 were considered significant for CCLE analyses. Log fold change (logFC) calculations were based on subtraction of average expression values of siRNA-CN controls from that of siRNA-1 treatment group.

### Comparisons with nutlin-3a dataset for TP53 induction of TP53 targets

A list of TP53 targets was retrieved from a recent study [42, 43] in which nutlin-3a treatment was used to determine TP53 mediated transcriptional changes in MCF7 cells using both RNA-seq and ChIP-seq results. These TP53 targets corresponded to 187 probesets among the jetset probesets of HGU-133 Plus 2. Expression profiles of probesets in this selected gene list were extracted from CHRNA5 RNAi microarray data to compare with the respective logFC values in the nutlin-3a dataset.

### Topoisomerase inhibitor expression data (GSE19638) reanalysis

Raw expression data [44] were normalized using *rma* [37]. DOXO and SN38 (a CPT derivative) samples were separately normalized with untreated samples and jetset best probeset expression data were analyzed using *limma* [38, 39].

### CCLE for CHRNA5 co-expression analysis

Cancer cell line encyclopedia (CCLE) GCRMA normalized expression dataset was downloaded from Broad Institute website [45]. CCLE data annotation was based on the customized annotation of Affymetrix HG-U133 Plus 2. Mapping file of CCLE customized probes to Affymetrix HG-U133 Plus 2 was downloaded from Broad Institute website and common jetset best probesets [38] were used for further analyses. In case of two CCLE best probesets mapping to one HG-U133 Plus 2 probeset, the one exhibiting a greater coefficient of variation in its expression was chosen. Pearson correlation coefficients were calculated using stat package in R [46]. In addition, a filtering criterion was introduced to exclude cell lines showing a normalized expression value of less than 5 for the gene of interest (excluding CHRNA5) before performing correlation analysis.

### Comparative transcriptomics with A549 CHRNA5 siRNA dataset

Raw cel files of GSE101979 [47] were downloaded from GEO and *rma* normalized using *affy* [37] package and differential expression analysis was performed using *limma* [39] package in R. Probesets were annotated using Biomart [48] R package. For genes having multiple probesets, a single probeset with the minimum p-value was considered for further analysis. Functional analysis was performed using DAVID [49].

### Analysis of TCGA and METABRIC datasets for correlation of CHRNA5 with TP53 mutation status and DDR signaling

For TCGA data analysis, sample data containing RSEM normalized values along with .maf files for mutation status were obtained from FireBrowse [50]. Log2 transformed values (RSEM+1) were used for further analysis. METABRIC normalized mRNA expression and TP53 mutation data were downloaded from European Genome Archives with accession ID EGAS00000000122 [51]. Plotting of CHRNA5 expression values for TP53 wildtype or mutant tumors was performed using a customized Matlab code. DDR genes (n = 60) were obtained from Lin et al. (2016) [52]. For hierarchical clustering, the expression datasets were normalized with their sample specific mean values. Hierarchical clustering was performed for expression levels of selected DDR genes along with that of CHRNA5. For TCGA dataset, a correlation-based distance matrix and complete linkage were used while for METABRIC dataset, Ward’s inner squared distance linkage was used. Genes clustered together with CHRNA5 were selected and clustered again using Ward’s inner squared distance linkage. In this cluster, samples were annotated also with the TP53 mutation status.

### Correlation between CHRNA5, CHEK1 and genomic alterations

cbioportal.org web-based tool was used to obtain CHRNA5 and CHEK1 mRNA expression values, from CCLE, METABRIC and TCGA datasets, along with the fraction of total genomic alterations where available [53]. For CCLE and METABRIC, log2 transformed microarray data, and for TCGA, log2 transformed RSEM+1 data were used for analyses. Correlation as well as linear regression for CHRNA5 expression values were calculated in Graphpad (Prism 6).

### Functional and chromosomal enrichment analyses in the CHRNA5 siRNA transcriptome

We used GSEA (Gene Set Enrichment Analysis) for functional annotation for KEGG pathways using MSigDB [40, 54]. Jetset best probesets [38] of *rma* normalized microarray data were used for the enrichment analysis with default options. As suggested by the developers, FDR q value <0.25 was used as a threshold.

In addition, DAVID (version 6.8) [49] was used for KEGG pathway analysis separately for the upregulated and downregulated probesets obtained from the merged CHRNA5 RNAi microarray dataset and the significant CCLE correlation profile.

R package *Category* [55] was used for chromosome enrichment analysis of CHRNA5 correlated genes from CCLE breast cell lines (r >0.3 and p <0.05) which were also downregulated by CHRNA5 siRNA-1 (logFC < -0.5 and p <0.05).

### MTT assay

2x10^3^ MCF7, BT-20 and MDA-MB-231 cells were seeded in 96-well plates in triplicates and next day cells were exposed to one of the siRNAs or siRNA-CN molecules as previously described. For drug sensitivity measurements, camptothecin (CPT; C9911-100MG, Sigma Aldrich) and doxorubucin (DOXO; 592S-Cell Signalling) were also added. A dose response range was first determined using MCF7 cells at doses between 0 and 2 μM drug treatments. After optimization, cells were exposed to five different doses (0.25μM, 0.125μM, 0.06μM, 0.03μM and 0.015μM) of either CPT or DOXO or to only DMSO control in the presence or absence of RNAi (siRNA or siRNA-CN) molecules. At the end of 72h incubation, MTT (3-(4,5-Dimethyltiazol-2-yl)-2,5-Diphenyltetrazolium Bromide) assay was performed using Vybrant MTT Cell Proliferation Assay Kit (V-13154, Invitrogen) based on manufacturer’s instructions. Color absorbance was measured using Microplate Spectrophotometer (μQuant, Biotek) at a wavelength of 570nm. Results were normalized against the mean of measurements from at least three replicas not exposed to any treatment.

### BrdU assay

After 72h of siRNA-1 and siRNA-CN treatment, MCF7 cells were exposed to 5-bromo 2-deoxyuridine (BrdU) (B5002, Sigma Aldrich) for 2h and fixed with cold ethanol. Cells were stained with anti-BrdU (5252, Cell signaling) followed by anti-mouse AlexaFluor-555 (#4409, Cell Signaling) and were then counterstained with blue fluorescent 4-6-diamidino-2-phenylindole (DAPI) for 10 min. Images for each treatment were taken from at least four different fields of the duplicates. Proliferation rate was calculated manually and blindly as the ratio of BrdU-positive nuclei (red) to total cell count (DAPI-positive nuclei, blue). Images were obtained using a fluorescent microscope (Zeiss-Axioimager A1 Carl, Germany).

### PI-FACS

2x10^5^ MCF7 cells seeded in 6-well plates and next day they were treated either with 10nM siRNA-1 or siRNA-CN in triplicates and incubated 72h at 37°C. Prior to staining procedure, cell suspension and PBS rinse together with the detached cells after trypsinization were collected into the same falcon. After washing with PBS, cells were fixed in 70% ethanol and stored at +4 ^o^C. 72h post-fixation, cells were washed twice and stained with PI solution containing 50ug/ml PI (P4864, Sigma-Aldrich), 0.1mg/ml RNase A (34388, Serva), and 0.05% Triton X-100 (T8787) in PBS. Samples were then incubated at 37°C for 40 minutes. Stained cells were analyzed in BD Accuri C6 using Cell Cycle Analysis 1.8 Software.

### Western blot (WB) analysis

72h or 120h treatment of siRNA-1 and 72h of siRNA-2 and -3 in MCF7 cells, and 72h treatment of siRNA-1 in BT-20 and MDA-MB-231 cells were performed in 6-well plates, as described previously. Cells were scraped with using freshly prepared RIPA buffer (NaCl, Tris HCl, NP-40, 10% SDS, PI and PhosSTOP). Total protein concentration was determined with BCA Protein Assay Reagent Kit (Thermo Scientific, USA,23227). Proteins were denatured with 4X Loading Dye (Biorad, USA, 161–0747) at 95 ^o^C for 5 minutes. Proteins were run in 10% SDS-PAGE (161–0183, Bio-Rad Acrylamide Kit) and then transferred onto PVDF membrane (3010040, Roche) by semi-dry transfer method. The membrane was then blocked in 5% BSA-Tris-buffered saline with Tween-20 (0,2%) (TBST) for 1h at room temperature after which incubated at 4°C overnight with either of the following primary antibodies: anti-Cholinergic Receptor Nicotinic Alpha 5 (CHRNA5), a monoclonal antibody with an affinity to a common region of all CHRNA5 isoforms and with the highest recognition of the transmembrane spanning and functional variant, CHRNA5_v2 (Monoclonal rabbit, EPR5395, expected band size at 50–75 kD; Abcam); anti-Glyceraldehyde-3-Phosphate Dehydrogenase (GAPDH; Monoclonal mouse, sc-47724, Santa Cruz Biotechnology); anti-Fas cell surface death receptor (FAS; Monoclonal Mouse, 8023, Cell Signaling); anti-Phospho-RB (Ser807/811) (pRB; Polyclonal rabbit, 9308, Cell Signaling, a kind gift from Dr. Sreeparna Banerjee); anti-BAX (monoclonal rabbit, 5023, Cell Signalling); anti-BCL2 (Monoclonal rabbit, 2870, Cell Signalling); anti-pCHEK1(Ser345) (Polyclonal rabbit, 2348, Cell Signalling); anti-Total Chek1 (Mouse monoclonal, sc-8408, Santa Cruz Biotechnology); anti-BID (Polyclonal rabbit, 2002, Cell Signalling); anti-Total CASP7 (Polyclonal rabbit, 9492, Cell Signalling; a kind gift from Dr. İhsan Gürsel); anti-γH2AX (Ser139) and anti-CASP7 (Monoclonal mouse, 9718 and monoclonal rabbit, 8438, Cell Signalling respectively; kind gifts from Dr. Özgür Şahin) at a dilution of 1:1000. Membranes were incubated with secondary antibodies, i.e., either anti-mouse (7076, Cell Signaling) or anti-rabbit (7474, Cell Signaling) for 1h at room temperature and washed with TBST. Specific proteins were detected using ECL Plus Western Blotting Detection System (RPN 2232, GE Healthcare) and captured on MXBE-Film (771 0783, Carestream) and Amersham (TM) Imager 600. For densitometry analyses the intensity values of target protein and the reference GAPDH from each panel of experiments were measured using ImageJ program [56]. The area under curve for each band was then calculated separately for each panel as percentages before normalizing each band to its own GAPDH percentage followed by normalization of all bands in a panel to the selected control group, i.e., treatments with siRNA and siRNA-CN in MCF7 cells: siRNA-CN (10nM) for siRNA-1 and siRNA-CN (50nM) for siRNA-2 and siRNA-3; treatments with siRNA alone and/or with drugs in MCF7 cells: for CPT, DSMO+siRNA-CN and for DOXO first DOXO+TR then DMSO+siRNA-CN; treatments with siRNA alone and/or with drugs in BT-20 and MDA-MB-231 cells: DMSO+siRNA-CN. The resulting values were logarithmically transformed at base 2 and statistically analyzed as described in text and visualized using Discriminant Function Analysis (DFA) [57].

### Statistical analyses

Graphpad (Prism 6) was used to draw figures and statistical analyses. Experimental data from RNAi treatments (RT-qPCR ΔΔCt [58], relative cell viability, counts and percentages) were analyzed using One-Way ANOVA and multiple test comparisons (Tukey HSD test). 120h siRNA-1 RT-qPCR results, having two replicates for siRNA-1 and one for siRNA-CN, were analyzed using one-sample t-tests. MTT results were analized using One-Way-ANOVA and multiple test comparisons (Tukey HSD test). Western blot densitometry measurements were analyzed depending on the panel, using appropriate controls, via either Student’s t-tests, One-Way or Two-Way ANOVAs followed by uncorrected Fisher’s LSD tests (simple effects tests for siRNA treatment and main effects for drug treatments) unless specified differently in the figure legends.

## Results

### Bioinformatics analysis of CHRNA5 expression in breast cancer cell lines and tumors

**Fig 1:**
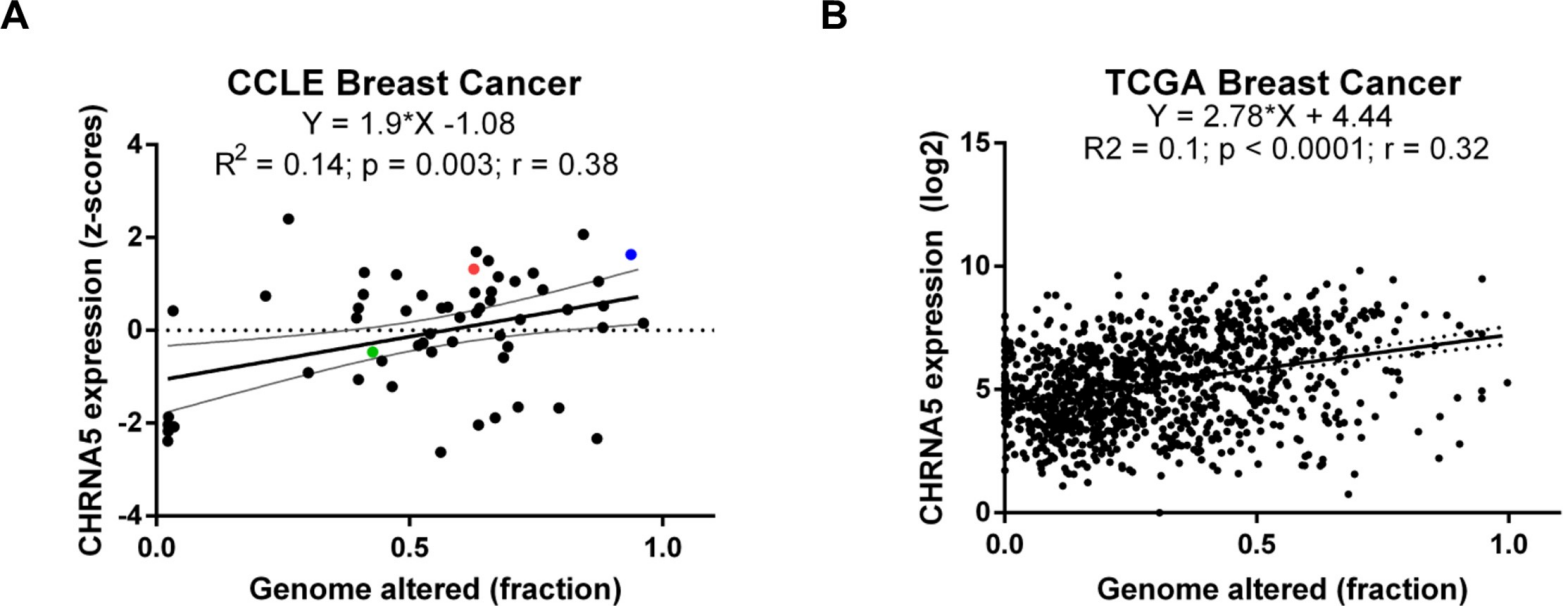
The correlation between CHRNA5 and the fraction of total genomic alterations. **A-B.** Linear regression and correlation coefficients between the CHRNA5 expression level and the fraction of genomewide alterantions in CCLE (A) and TCGA (B) datasets. The colors blue, red, and green, respectively, indicate MCF7, BT20, and MDA-MB-231 cell lines in (A).

We calculated the correlation coefficient between CHRNA5 expression level and the fraction of genome-wide alterations obtained for breast cancer cell lines and breast tumors, respectively, using CCLE and TCGA datasets. This allowed us to examine the variability in expression levels of CHRNA5 in cell lines and tumors while showing a significant association with genomic instability (Fig 1). Among the breast cancer cell lines used in the present study MCF7 exhibited high expression values and had an amplified CHRNA5 locus while the expression of BT20 was higher than that of MDA-MB-231 based on the CCLE dataset (Fig 1A).

### CHRNA5 RNAi model in MCF7 breast cancer cells

**Fig 2:**
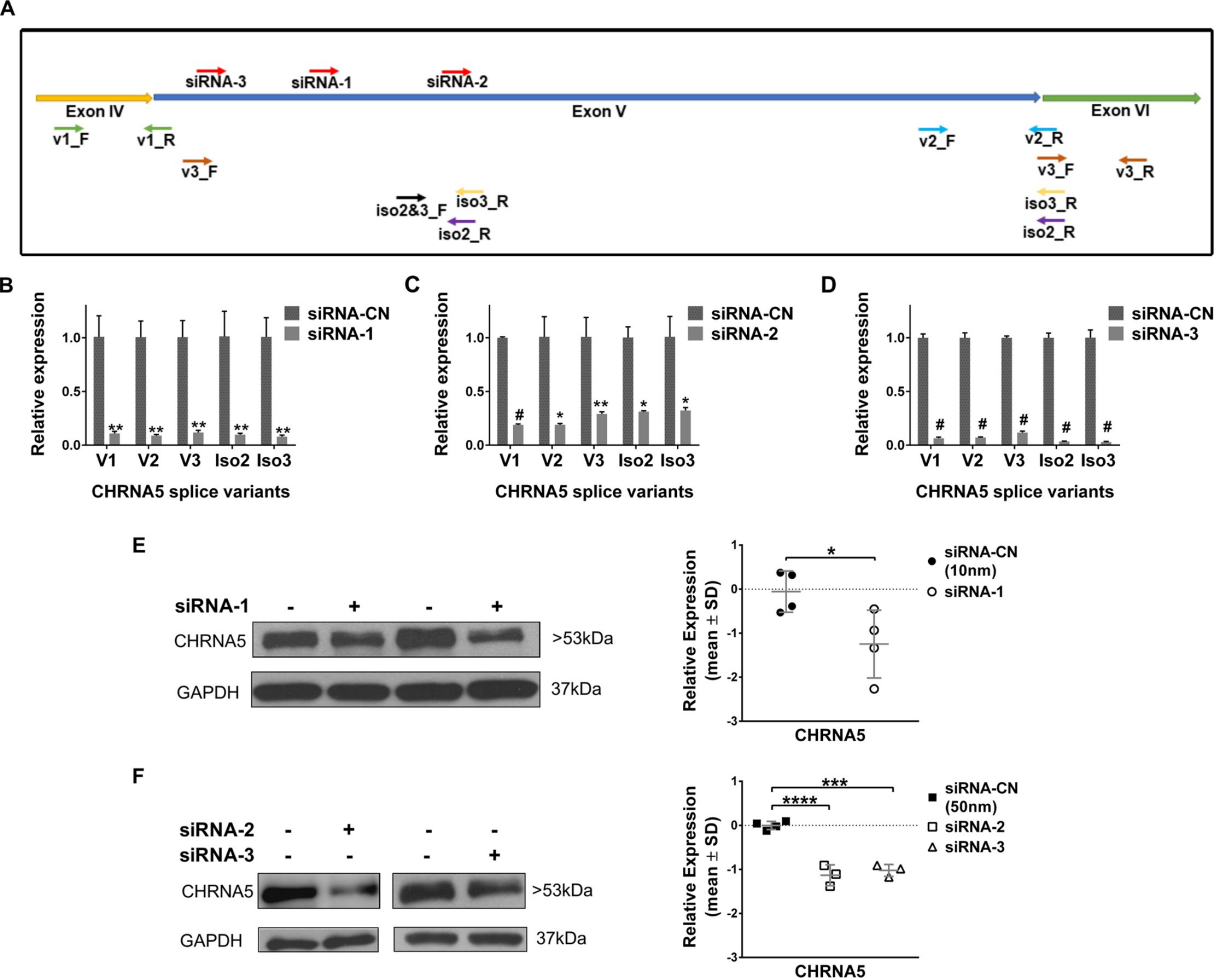
Downregulation of CHRNA5 expression by RNAi. **A.** Schematic representation of target sites of siRNA molecules and primers for CHRNA5 isoforms. **B-D.** Downregulation of CHRNA5 isoforms in MCF7 cells upon transient transfection with siRNA-1 (10nM) (B), siRNA-2 (50nM) (C), and siRNA-3 (50nM) (D) in comparison with the corresponding siRNA-CN at 72h of treatment (n = 2 per group). Student’s t-test was applied. **E-F.** Depletion of CHRNA5 expression at protein level (Western Blotting together with densitometry analyses, loading control, GAPDH) with siRNA-1 (10nM) (n = 4 per group; Student’s t-test) (E), siRNA-2 and siRNA-3 (n = 4 for siRNA-CN; n = 3 for siRNA-2 and siRNA-3; One-Way ANOVA followed by Tukey’s HSD tests) (F). (*: p < 0.05; **: p < 0.01; ***, #: p < 0.001; **** p < 0.0001).

Application of all three siRNAs for 72h against CHRNA5, whose target sites were shown in Fig 2A, significantly reduced the gene and isoform specific mRNA expression of CHRNA5 (see for primers Fig 2A and S1 Table) in MCF7 cells (Fig 2B–2D). At the protein level, significant depletion of CHRNA5 was observed with all three siRNA molecules in MCF7 cells, supporting the CHRNA5-specific targeting of RNAi (Fig 2E and 2F). Moreover, application of 120h siRNA-1 also resulted in strong downregulation of CHRNA5 both at the transcript and protein level (S1A–S1B Fig).

### Signaling pathways modulated by CHRNA5 RNAi

**Fig 3:**
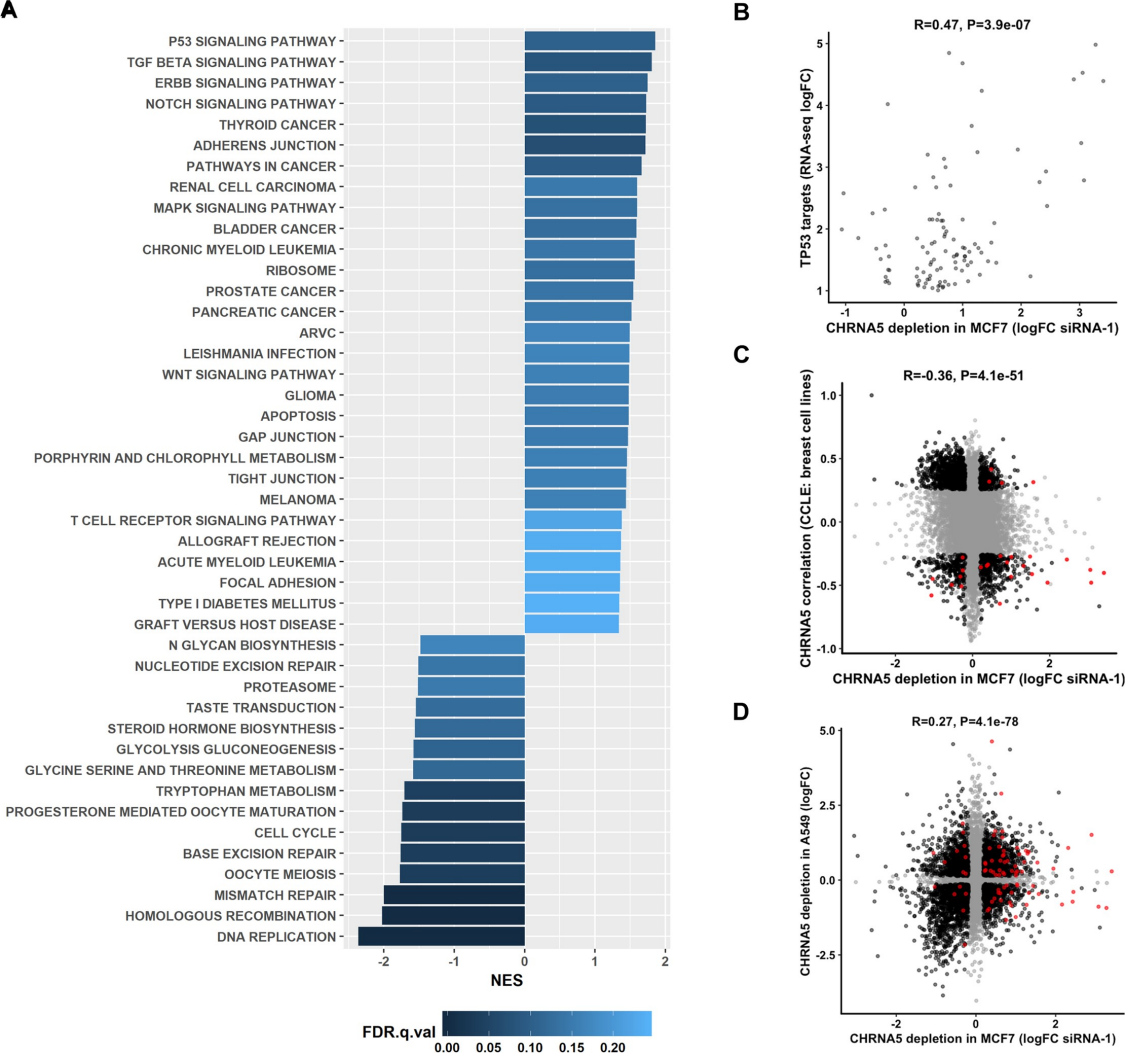
Functional analysis and comparative transcriptomics of CHRNA5 RNAi data. **A.** Normalized Enrichment Scores (NES) of GSEA analysis of microarray results (FDR q value < 0.25). **B.** Correlation between logFC values of nutlin-3a and siRNA-1 treated MCF7 cells for TP53 targets (confirmed by chip-seq, i.e., chip-seq peak = yes) significantly moduled by siRNA-1. **C.** Correlation profile of CCLE breast cancer cell lines versus CHRNA5 RNAi logFC after filtration. **D.** Correlation between CHRNA5 RNAi logFC values in MCF7 with CHRNA5 RNAi logFC values in lung cancer cell line A549. For C and D, genes significant in both categories are colored black; genes insignificant (or significant in one type of data) are shown in gray; and TP53 targets are shown in red.

Affymetrix HG-U133 Plus 2 arrays were used for profiling the expression changes upon application of CHRNA5 siRNA-1 in comparison with that of siRNA-CN in MCF7 cells. Quality control analyses revealed that the microarrays were of high quality (n = 4; more than 90% correlation among all arrays; percent present values of arrays: 41.89%, 42.39%, 42.21%, and 42.23%; average background: 45.78, 44.41, 44.68, and 46.38, respectively). *Limma* analysis with jetset (version: 3.1.2) best probesets [38] and multiple test correction using Benjamini-Hochberg (BH) method resulted in a total of 4323 differentially expressed probesets (2265 downregulated and 2058 upregulated; p < 0.05). Functional annotation using GSEA and MSigDB [40, 49, 54] for KEGG pathways demonstrated that the upregulated probesets were enriched in TP53 signaling and pathways involved in cancer as well as adherens junction while the downregulated ones were enriched in functions such as DNA replication, cell cycle, and DNA repair mechanisms (Fig 3A).

We then compared TP53 target probesets modulated by TP53 pathway activator nutlin-3a treatment (also validated by Chip-seq) in MCF7 cells with the CHRNA5 RNAi expression profile. We identified 106 out of 187 TP53 target probesets as being significantly modulated also by the siRNA-1 in MCF7 cells (p < 0.05). Most of the targets in the TP53 target list were upregulated after the siRNA-1 treatment as in nutlin-3a while there was a significant positive correlation between these two datasets (r = 0.47, p = 3.9E-07) (Fig 3B). Our results showed that TP53 target genes were indeed significantly induced by CHRNA5 RNAi in the MCF7 cell line possessing a wild type TP53. Induction of TP53 target genes might indicate cell cycle arrest, apoptosis or DNA damage, which have been addressed in the next sections.

Comparison of the co-expression profile of CHRNA5 expression across 59 CCLE breast cancer cell lines with the logFC values from CHRNA5 RNAi profile revealed a highly significant negative correlation and association (r = -0.33, p = 3.8E-48). Filtration of cell lines exhibiting no or low expression of the gene of interest slightly improved the strength of this association (r = -0.36, p = 4.1E-51; Fig 3C). This effect was more pronounced in a group of genes downregulated by the siRNA-1 and positively correlated with CHRNA5 in CCLE (Fig 3C). Although a significant negative correlation between datasets was observed, a considerable number of genes also exhibited deviation from the above-mentioned trend (i.e., negatively correlated with CHRNA5 while being downregulated after the siRNA-1 treatment and vice-versa). Functional annotation using KEGG pathways revealed that these shared (siRNA-1 downregulated and CCLE positively correlated with CHRNA5) genes were primarily involved in cell cycle, DNA replication and repair related processes (S2 Table). Our findings supported that transcriptomic changes associated with the CHRNA5 siRNA-1 may not be specific to MCF7 cells and could be extended to a large set of breast cancer cell lines. Shared set of genes positively correlated with CHRNA5 expression in CCLE while being downregulated by the siRNA-1 (CCLE filter: p < 0.05 and r > 0.3; siRNA-1 filters: logFC < -0.5 and p < 0.05) were also used for the chromosome enrichment analysis. The results indicated that genes located on chromosome 15 were highly coexpressed with CHRNA5 (also located on 15q25) (Table 1 and S3 Table).

We also performed a correlation analysis between the expression profiles obtained from breast MCF7 and lung A549 cell lines and found a significantly positive correlation (Fig 3D) while the TP53 targets exhibited common as well as cell-line specific changes. The functional annotation of commonly modulated probesets in the two datasets revealed genes downregulated in both datasets were enriched in DNA replication, cell cycle and DNA repair related pathways (S4 Table).

**Table 1:** Chromosome enrichment (Top 5) of CHRNA5 modulated genes (depleted by the siRNA-1 in MCF7 and positively correlated with CHRNA5 in CCLE).

ChrMapID	PValue	OddsRatio	ExpCount	Count	Size
15	6.06E-04	2.35	9.49	21	474
15q22.31	2.21E-03	8.24	0.56	4	28
15q26.1	2.53E-03	7.91	0.58	4	29
1p34.1	5.07E-03	6.38	0.7	4	35
15q22	7.45E-03	4.42	1.22	5	61

### Microarray validation studies

**Fig 4:**
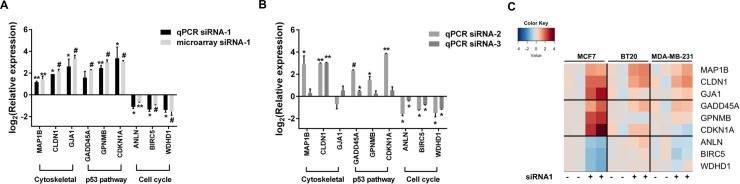
RT-qPCR validation of CHRNA5 RNAi microarray data. **A.** RT-qPCR validation of selected genes modulated by siRNA-1 in MCF7 cells. **B.** RT-qPCR validation of selected genes in siRNA-2 and siRNA-3 treated MCF7 cells. **C.** Heatmap of RT-qPCR analysis results of selected genes in MCF7, BT20 and MDA-MB-231 in the presence of siRNA-CN or siRNA-1. For siRNA-1, and siRNA-2 and -3 treatment RT-qPCR results, student’s t-tests in comparison with their corresponding siRNA-CN group, i.e., 10nM for siRNA-1 and 50nM for siRNA-2 and -3, and for microarray data *limma* were used. (*: p < 0.05, **: p < 0.01, #: p < 0.001). (n = 2 per group for all cell lines).

We next validated the microarray-enriched pathways using RT-qPCR based on independent RNAi experiments with siRNA-1 at 72h in MCF7 cells (Fig 4A). RT-qPCR analysis indicated a significant increase in mRNA expression of genes related to structural and cell junction proteins, e.g., GJA1, MAP1B, and CLDN1 (Fig 4A). Expressions of multiple TP53 targets, i.e., GADD45A, GPNMB, and CDKN1A, were also increased (Fig 4A). The tumor suppressor gene CDKN1A was one of the most upregulated while oncogenic cell cycle genes such as WDHD1 and BIRC5 were among the downregulated genes (Fig 4A). ANLN, another gene associated with positive regulation of cell cycle, also showed a significant reduction of expression in both the microarray and RT-qPCR experiments (Fig 4A). Results from the exposure to CHRNA5 siRNA-2 and -3 largely supported the robustness of RNAi effects (Fig 4B). In addition, the observed effects in 72h lasted through 120h of siRNA-1 exposure (S1C Fig). Expressions of genes affected by RNAi in MCF7 cells along with the expression of *CHRNA5 isoform_v1* were also analyzed in the TP53 mutant cell lines, MDA-MB-231 and BT-20, upon siRNA-1 application by qRT-PCR (Fig 4C; S2A Fig). CHRNA5 mRNA levels were significantly reduced in these two cell lines although the heatmap indicated milder downstream effect of CHRNA5 RNAi in BT-20 and MDA-MD-231 than in MCF-7 cells (Fig 4C). Univariate statistical analysis showed that CHRNA5-associated expression changes in BT-20 were only significant in cytoskeletal representatives (MAP1B, CLDN1). CHRNA5 RNAi induced effects on MDA-MB-231 and BT-20 cell lines (both being TP53 mutant) were not significant for the known TP53 targets (e.g., CDKN1A, GPNMB, GADD45A; Fig 4C) confirming the requirement of a wildtype TP53 for induction. Our findings suggested some effects of CHRNA5 RNAi on gene expression could be based on TP53 status of the cell lines.

### Effects of CHRNA5 RNAi on cell viability, proliferation, apoptosis and DNA damage

**Fig 5:**
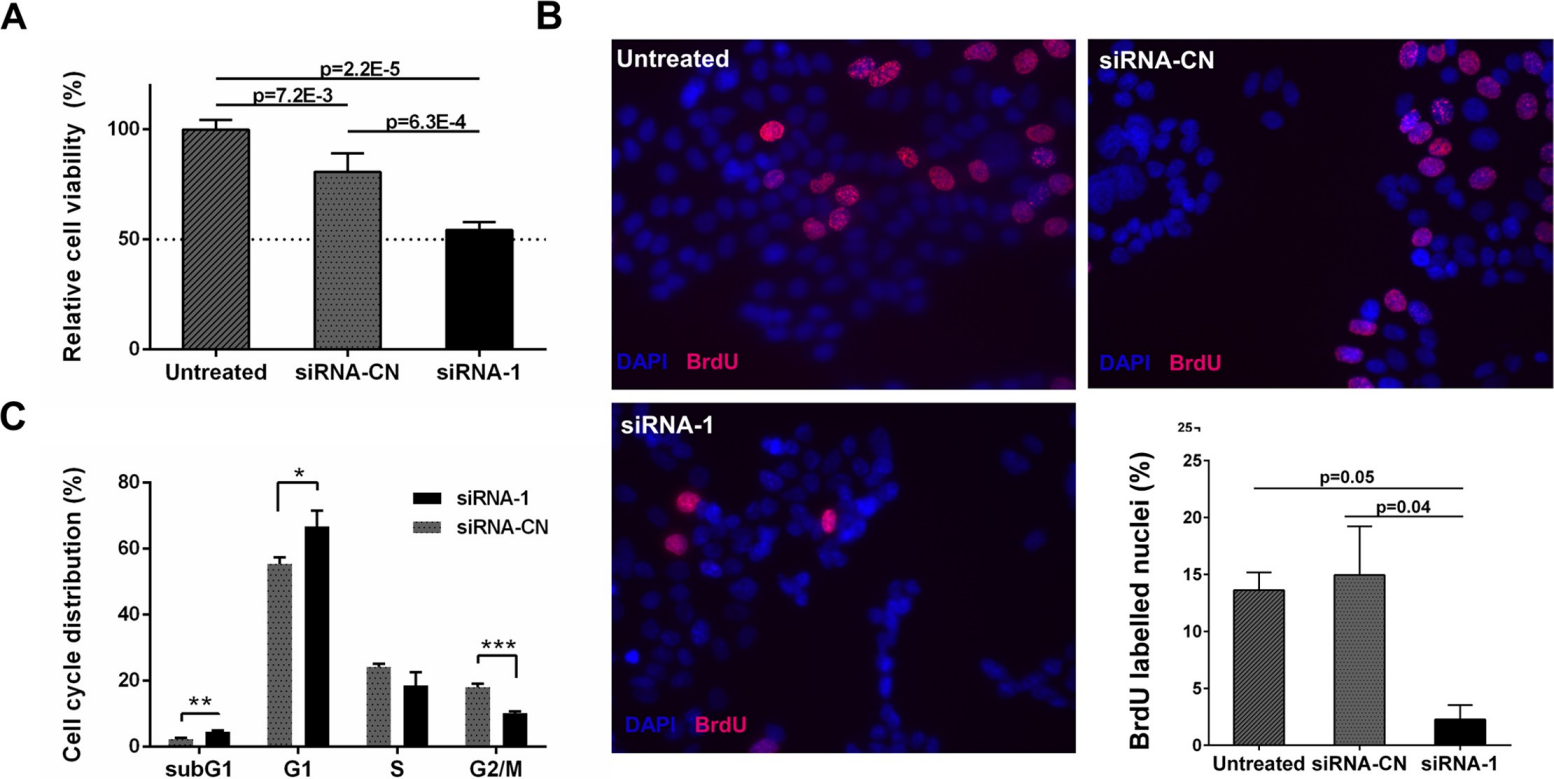
The effect of CHRNA5 depletion on cell cycle arrest and apoptosis in MCF7 cells. **A.** Relative cell viability measured with MTT assay (n = 3 per group). **B.** BrdU incorporation assay (n = 2 per group). BrdU positive (pink) and DAPI (blue) labelled cells were shown in representative images of treatments. **C.** PI staining and FACS analysis (n = 3 per group). (*: p < 0.05, **: p < 0.01, ***: p < 0.001).

Transcriptional studies of CHRNA5 RNAi applications indicated a reduction in cell viability and cell cycle arrest. For validation, we performed MTT assays with CHRNA5 siRNA-1 in comparison to siRNA-CN (10nM) showing approximately a 30% reduction in MCF7 cell viability (Fig 5A). MTT assay was repeated using three different siRNAs against CHRNA5 at 72h of exposure validating the antiproliferative effects of CHRNA5 depletion (S2B Fig). These findings also suggested that siRNA molecules used in this study exhibited target specific siRNA-induced cytoxicity in MCF7 cells [59]. Although we could downregulate CHRNA5 expression in BT-20 and MDA-MB-231 cells (S2A Fig), effects of CHRNA5 RNAi on cell viability depend on the cell line (S2C–S2D Fig).

BrdU incorporation assay using CHRNA5 siRNA-1 in MCF7 cells showed that siRNA treated samples had a lower percentage of BrdU-positive cells, indicating a reduction in DNA synthesis rate and leading to the cell cycle arrest (Fig 5B). These results were validated by propidium iodide (PI) staining showing siRNA-1 treated MCF7 cells were enriched at the G1-phase with a corresponding reduction at the G2-M phase (Fig 5C).

**Fig 6:**
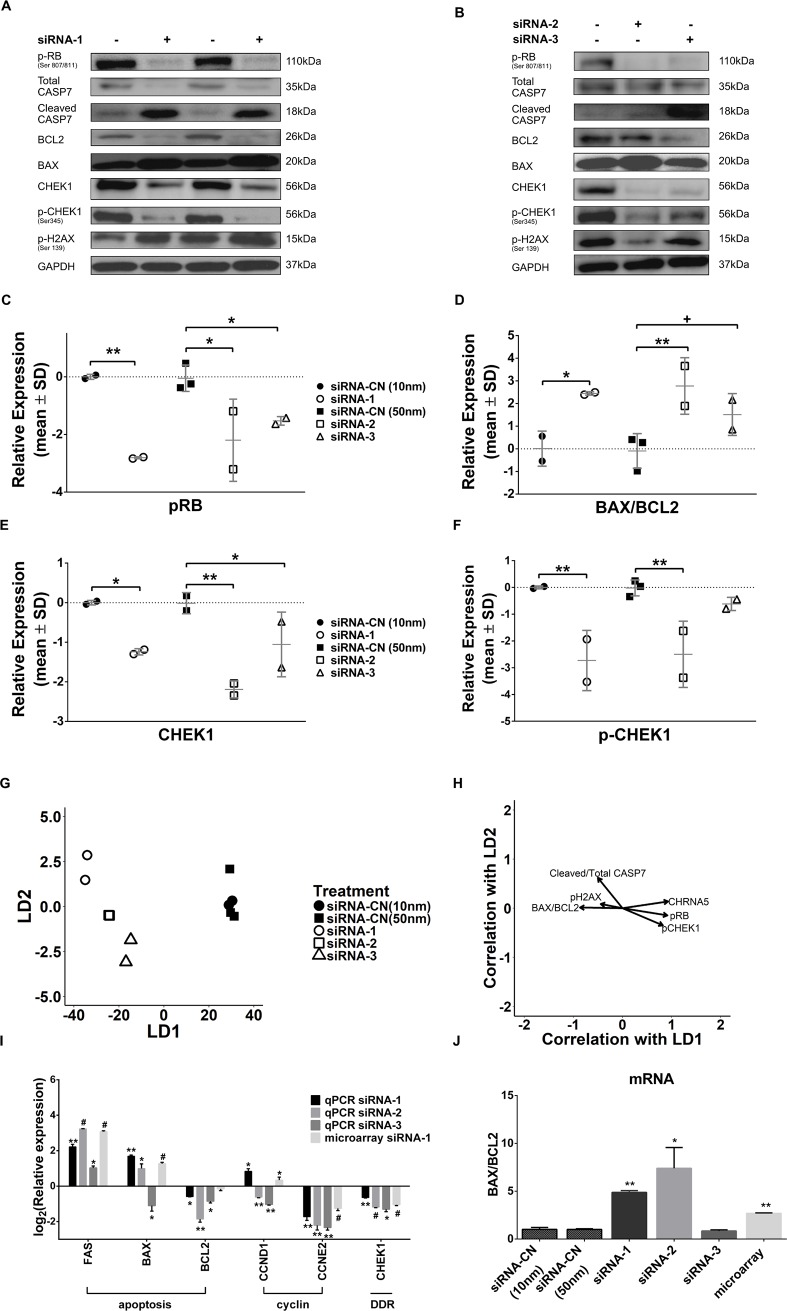
Western blotting and RT-qPCR analyses relevant to effects of RNAi on cell cycle, apoptosis and DDR in MCF7 cells. **A-B.** Western Blot results for pRB, total CASP7, cleaved CASP7, BCL2, BAX, total CHEK1, pCHEK1, and pH2AX in siRNA-1 (A) and siRNA-2 and siRNA-3 (B) treated MCF7 cells. **C-F.** Densitometry measurements of pRB (C), BAX/BCL2 ratio (D), total CHEK1 (E), and pCHEK1 (F). One-Way ANOVA was used in comparison with corresponding control groups, siRNA-CN (10nM) vs. siRNA-1 and siRNA-CN (50nM) vs. siRNA-2 and -3. **G.** DFA plot for control and RNAi treatment groups. **H.** Vector weights of variables and their correlation with LD1 and LD2 of DFA. (n = 2 per group for siRNA-CN (10nM) and siRNA-1; n = 3 per group for siRNA-CN (50nM) and siRNA-2 and -3). **I.** RT-qPCR results of FAS, BAX, BCL2, CCND1, CCNE2, and CHEK1 in siRNA-1-3 treated MCF7 cells (n = 2 per group). **J.** BAX/BCL2 ratio from RT-qPCR results (n = 2 per group). One-way ANOVA followed by Tukey’s multiple test correction was used. (+: p < 0.1, *: p < 0.05, **: p < 0.01, #: p < 0.001).

Since there was also a significant increase in the percentage of sub-G1 cell population, we examined the expressions of selected cell cycle and apoptosis related proteins by Western Blotting for all three siRNAs (Fig 6A and 6B). Although microarray data did not indicate a change in RB mRNA levels (i.e., logFC -0.077, p = 0.37), phosphorylation of RB protein was reduced in 72h and 120h siRNA-1 treated cells (Fig 6A and 6C; S1D–S1E Fig). This finding was also confirmed using siRNA-2 and siRNA-3 molecules (Fig 6B and 6C). Levels of cleaved CASP7, an effective apoptotic marker, were then observed to significantly increase in siRNA-1 treated MCF7 cells while total CASP7 levels decreased at 72h and 120h (Fig 6A; S1D–S1E and S3A Figs). The ratio of cleaved CASP7/total CASP7 was significantly altered for siRNA-1 only while there was no significant change with the siRNA-2 or siRNA-3 exposures (S3A Fig). Moreover, an increased BAX and decreased BCL2 protein levels in response to siRNA-1 and siRNA-2 at 72h of exposure further suggested induction of apoptosis by CHRNA5 depletion (Fig 6A, 6B and 6D). We also demonstrated that all siRNA molecules resulted in significant decreases in total CHEK1 while only siRNA-1 and -2 decreased phosphorylated CHEK1 (S345) at the protein level (Fig 6A, 6B, 6E and 6F). Exposure to 120h of siRNA-1 reduced pCHEK levels but not CHEK1 (S1D–S1E Fig). Previous studies in breast cancer as well as in leukemia and lymphoma have shown that CHEK1 inhibition can be characterized by an increased level of pH2AX (Ser139) [20, 21, 60]. However, our results indicated that CHRNA5 RNAi could not induce a consistent increase in phosphorylation of H2AX at 72h of exposure (Fig 6A and 6B, S3B Fig), even though the effect of siRNA-1 on pH2AX was significant at 120h of exposure (S1D–S1E Fig). To determine whether treatment and control groups could be discriminated from each other based on overall differences in protein expression (Fig 6A and 6B), we performed DFA and analyzed the vector correlations (Fig 6G and 6H). The apoptotic and DDR protein panel indicated that all groups were separated from each other while biological replicates from each group were placed together (Fig 6G). The CHRNA5 RNAi treatments were associated with increases in BAX/BCL2 and cleaved CASP7/total CASP7 ratios, and to a lesser degree pH2AX levels while pCHEK1, pRB and CHRNA5 levels decreased relative to controls, i.e., siRNA-CN (10 nM and 50nM). Linear Discriminant 1 (LD1) had more effect than LD2 in the separation based on the magnitudes of vectors (Fig 6G and 6H).

Next, we have tested whether the cell cycle inhibitory, apoptosis and DDR related effects that we observed at the protein level were also reflected at the mRNA level. Modulators of E2F signaling (e.g., CCND1 and CCNE2 that bind and activate CDK4/6 and CDK2, respectively) leading to phosphosylation of RB protein [61] were also studied. We observed a significant reduction in CCNE2 mRNA levels in all siRNA treatments supporting cell cycle arrest (Fig 6I) while CCND1 was significantly increased upon siRNA-1 and decreased by siRNA-2 and -3 treatments (Fig 6I). In our microarray data, we also observed a decrease in E2F1 (logFC = -0.43, p = 0.004), CDC25A (logFC = -0.76, p = 9.18E-05), CCNE1 (log FC = -0.56, p = 0.001), and CCNE2 (log FC = -1.26, p = 8.36E-06, further confirmed with RT-qPCR) but not in CCND1 (logFC = 0.34 p = 0.02, futher confirmed with RT-qPCR for siRNA-1). In addition to these, RT-qPCR analysis of BAX, BCL2, FAS mRNAs and calculation of BAX/BCL2 mRNA ratio supported the reduction in proliferation and induction of apoptosis (Fig 6I and 6J). The observed decrease in BAX by siRNA-3 treatment was not confirmed by Western blotting suggesting it can be transient. FAS protein level also significantly increased in siRNA-1 treated cells at 72h and 120h of exposure (S3C Fig). In MDA-MB-231 cell line, expressions of cyclin genes, CCND1 and CCNE2, decreased upon siRNA-1 treatment, however BAX to BCL2 ratio didn’t change in BT-20 and MDA-MB-231 cells (S3D–S3E Fig). Western blotting results and GSEA enrichment analysis of microarray data showed that depletion of CHRNA5 has led to significant alterations in DDR. Moreover, RT-qPCR analysis showed that CHRNA5 RNAi resulted in reduced CHEK1 mRNA levels in MCF7 cells (Fig 6I).

### Coexpression of CHRNA5 with DDR genes using TCGA and METABRIC datasets

**Fig 7:**
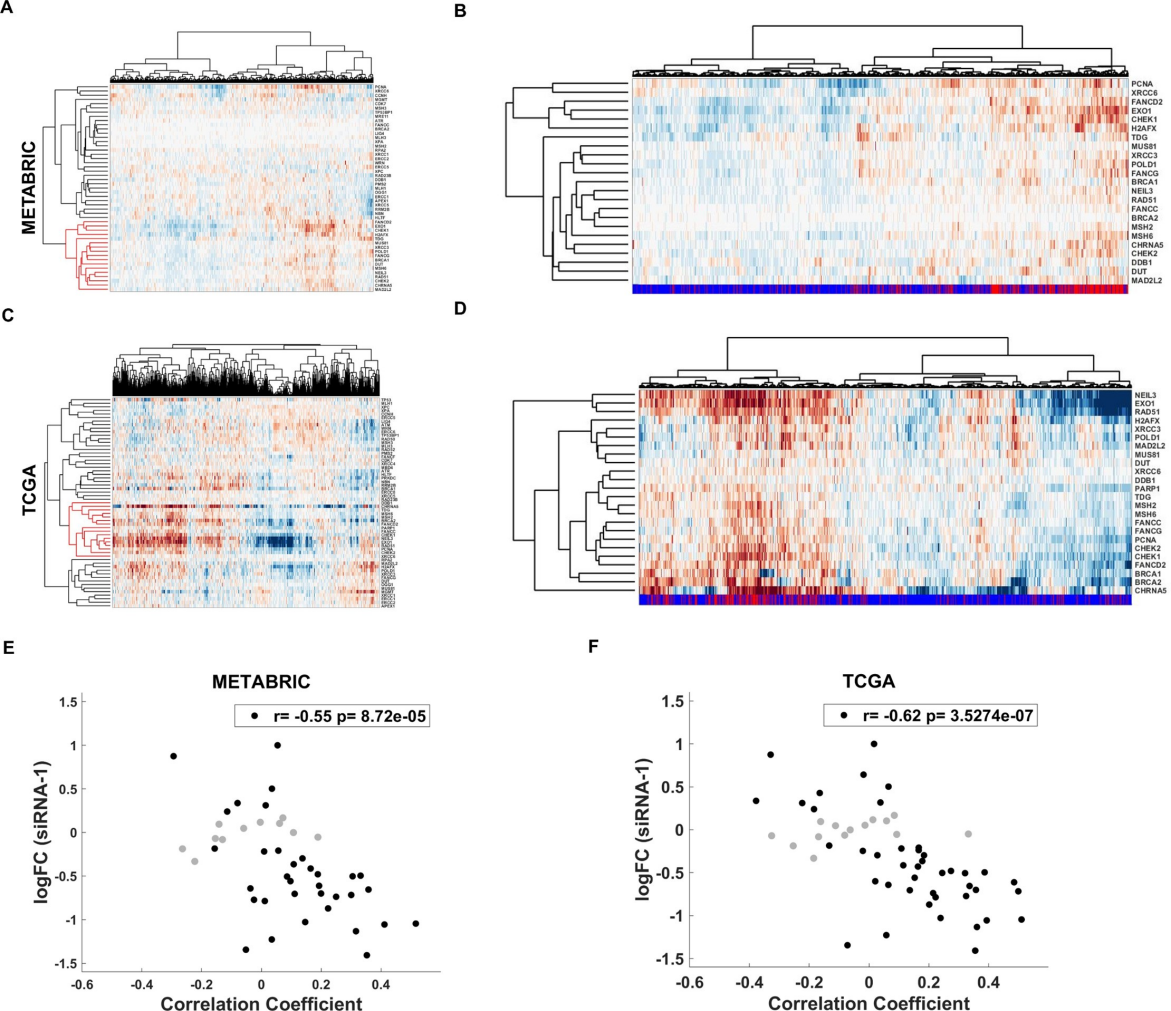
**Analysis of DDR genes in METABRIC and TCGA data A, C.** METABRIC (A) and TCGA (C) patient clustering with DDR genes and CHRNA5; red cluster includes CHRNA5. **B, D.** METABRIC (B) and TCGA (D) patient clustering for genes in red cluster shown in Fig 7A and 7C. Bottomline refers to TP53 mutation status: red, mutation; blue, wildtype. **E-F.** Correlation between logFC response to siRNA-1 treatment and correlation coefficient of CHRNA5 and DDR genes from METABRIC (E) and TCGA (F). Genes whose expression levels did not change significantly with siRNA-1 treatment are shown in gray (p < 0.05).

CHRNA5 RNAi transcriptional profile indicated that the most downregulated KEGG pathways were related to DNA replication and repair. RT-qPCR results for CHEK1 and Western blotting for CHEK1 and pCHEK1 indicated a deregulation of DDR in CHRNA5 depleted MCF7 cells. We then clustered *in silico* patient data based on expression of DDR genes obtained from Lin et al. (2016) [52] and observed that CHRNA5 clustered with a sub-group of DDR genes that included CHEK1, BRCA1, and RAD51 (see S5 Table for a full list) in both datasets (Fig 7A and 7C, METABRIC and TCGA, respectively). Expression of DDR genes clustering with CHRNA5 also exhibited a high association with TP53 status (Fig 7B and 7D) while CHRNA5 expression was significantly higher in samples with TP53 mutations (for CHRNA5: p = 1.26e-38 for METABRIC; and p = 1.58e-21 for TCGA, S4 Fig). In addition, we plotted CHRNA5 siRNA-1 logFC values against correlation coefficients between CHRNA5 and DDR gene expressions showing a highly significant negative correlation consistently in both datasets (Fig 7E and 7F).

### CHRNA5 as an inhibitor of CHEK1 and enhancer of drug sensitivity

**Fig 8:**
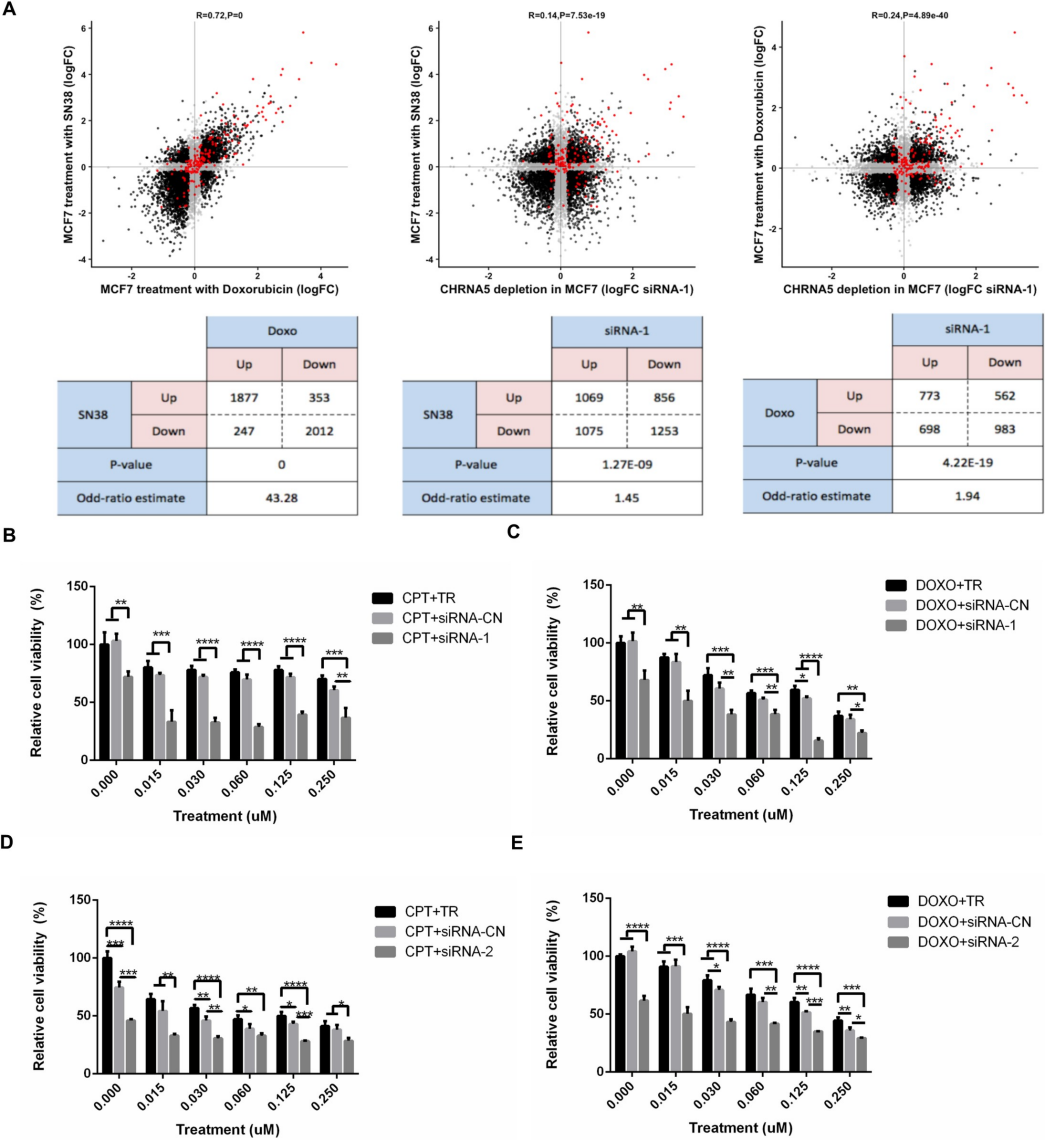
Association between CHRNA RNAi and topoisomerase inhibitors. **A.** Comparison of log fold changes (logFC) in response to DOXO and SN38, siRNA-1 and SN38, siRNA-1 and DOXO treatment in MCF7 (from left to right) and the corresponding statistical analysis results (Fisher’s Exact Test counts and p-value; bottom). Genes significant in both categories are colored black; genes insignificant (or significant in one type of data) are shown in gray; and TP53 targets are shown in red. **B-E** Relative cell viability for CPT and siRNA-1 (B), DOXO and siRNA-1 (C), CPT and siRNA-2 (D), and DOXO and siRNA-2 (E) treatments at increasing concentrations of CPT (B, D) and DOXO (C, E). One-Way ANOVA followed by Tukey’s multiple test correction were used for statistical analysis of MTT assays (*: p < 0.05, **: p < 0.01, ***: p < 0.001, ****: p < 0.0001).

CHRNA5 RNAi significantly reduced expression of multiple genes involved in DNA replication (Figs 3 and 4). Topoisomerase inhibitors camptothecin (CPT) and doxorubicin (DOXO), which stabilize the enzyme-DNA complex and prevent the DNA replication process, are widely used as cancer therapeutic agents [62]. Comparative transcriptomics revealed a very significant association between differentially expressed genes of CHRNA5 siRNA-1 treatment and those of topoisomerase inhibitors in MCF7 cells (GSE19638) [44] (Fig 8A; DOXO vs. SN38, r = 0.72, p = 0; MCF7 siRNA-1 vs. SN38, r = 0.14, p = 7.53 x 10^−19^; MCF7 siRNA-1 vs. DOXO, r = 0.24, p = 4.89 x 10^−40^). Mapping of TP53 targets on the scatterplots also demonstrated that this could be partly due to TP53 activation [42] (Fig 8A). We then tested whether MCF7 cells treated with CPT/DOXO alone at variable doses or together with CHRNA5 siRNA-1 affected relative cytotoxicity. An MTT panel using concentrations between 0–2 μM of CPT and DOXO indicated that CHRNA5 RNAi when used together with CPT/DOXO exhibited higher cytotoxicity at the 0.125–0.250 μM range (S5 Fig). Therefore, further analysis using a finer dose range with siRNA-1 (Fig 8B and 8C, for CPT and DOXO, respectively), and also with siRNA-2 (Fig 8D and 8E), confirmed the drug sensitizing effects of CHRNA5 RNAi in MCF7 cells.

**Fig 9:**
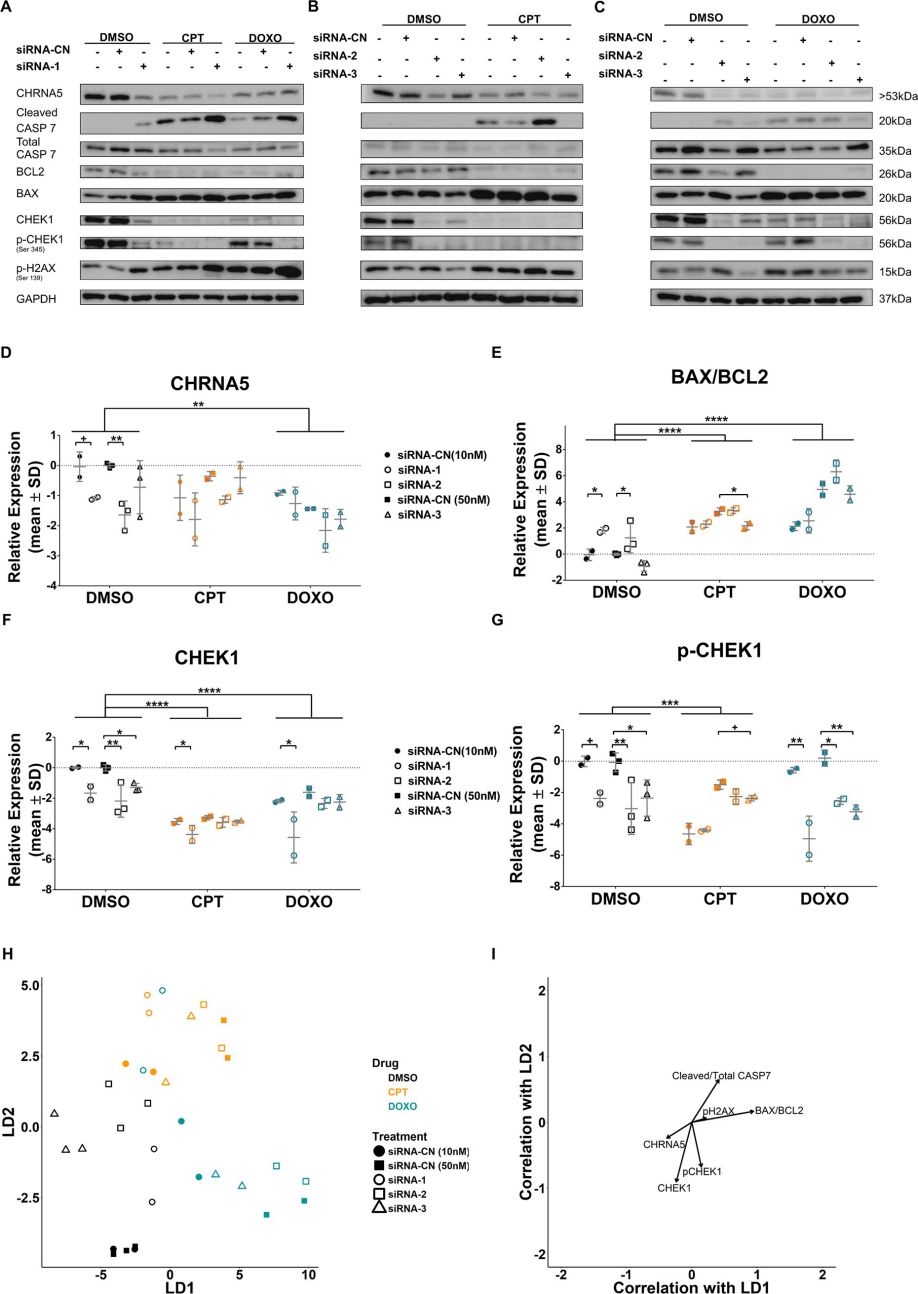
The effect of CHRNA5 siRNA in drug sensitivity. **A-C.** Western blot results in DMSO, CPT (0.125μM), and DOXO (0.125μM) treated MCF7 cells in the absence or presence of siRNA-1 **(A)**, siRNA-2, **(B)** and siRNA-3, **(C). D-G.** Two-Way ANOVA of densitometry measurements for CHRNA5 **(D),** BAX/BCL2 **(E)**, CHEK1 **(F)**, pCHEK1 **(G)**. **H**. DFA of densitometry measurements for control and treatment groups in A-C. **I.** Vector weights of variables and their correlation with LD1 and LD2 of DFA analysis. (n = 3 for DMSO siRNA-CN (50nM) and siRNA-2 and -3; n = 2 for other groups). (+: p < 0.1; *: p < 0.05, **: p < 0.01, ***: p < 0.001, ****: p < 0.0001).

We next tested whether CHRNA5 siRNA molecules used together with CPT/DOXO exhibited stronger effects than used alone on the levels of apoptosis and DDR related proteins, i.e., cleaved CASP7/total CASP7 and BAX/BCL2 ratios, total CHEK1 and phosphorylated CHEK1 as well as pH2AX using Western Blotting (Fig 9A–9C). We showed that CHRNA5 was significantly reduced by siRNA-2 and the reduction approached significance with siRNA-1 (Fig 9D). In addition, DOXO treatment significantly reduced CHRNA5 protein levels regardless of siRNA exposure (Fig 9D). CPT and DOXO treatments were highly effective in increasing BAX/BCL2 ratio when compared with the DMSO group (main effects) as BAX/BCL2 ratio increased by siRNA-1 and siRNA-2 treatments as previously shown in Fig 6 (Fig 9E). There was no additive effect of siRNA treatment on the increased BAX/BCL2 ratio apart from the effect of TOP inhibitors (Fig 9E). Total CHEK1 protein levels were significantly reduced by all three siRNAs in the DMSO treatment group (Fig 9F). CPT and DOXO treatments in the presence of siRNA-1 also resulted in a significant reduction in CHEK1 levels but not in the presence of siRNA-2 or siRNA-3 (Fig 9F). CPT and DOXO also had significant effects on overall total CHEK1 levels (main effects; Fig 9F). All three siRNAs were highly effective in reducing pCHEK1 protein levels under the DMSO treatment alone and also when given together with DOXO (Fig 9G). CPT in the presence of siRNA-3 also reduced the pCHEK1 levels (Fig 9G) but not in the presence of siRNA-1 or siRNA-2. With cleaved CASP7/total CASP7 ratio and pH2AX levels, the results did not indicate significant effects with siRNA molecules (S6A–S6B Fig). However, the ratio of cleaved CASP7/total CASP7 significantly increased with CPT and DOXO treatments and no further increase in the presence of siRNA molecules could be detected (S6A Fig). pH2AX significantly increased with DOXO treatment but an additional induction by siRNA molecules was not observed (S6B Fig). We again performed DFA on the control and treatment groups (Fig 9H and 9I) showing the level of discrimination posed by the WB panel protein changes (Fig 9H). The panel discriminated well between DMSO, DOXO, and CPT groups based on both LD1 and LD2. siRNA groups were distinct from their controls within the treatment group under DMSO treatment, yet this was less so in DOXO and CPT. siRNA-1 treatments when given with DOXO were closely placed next to the CPT treatments (Fig 9H) whereas other groups of DOXO remained distinct. DFA suggested that DMSO, CPT and DOXO had relatively distinct effects on the WB panel proteins (Fig 9H). Variables highly correlated with LD1 and LD2 clustered as in the Fig 6H yet the differential effects of CPT and DOXO could be observed based on the vector weights (Fig 9I).

**Fig 10:**
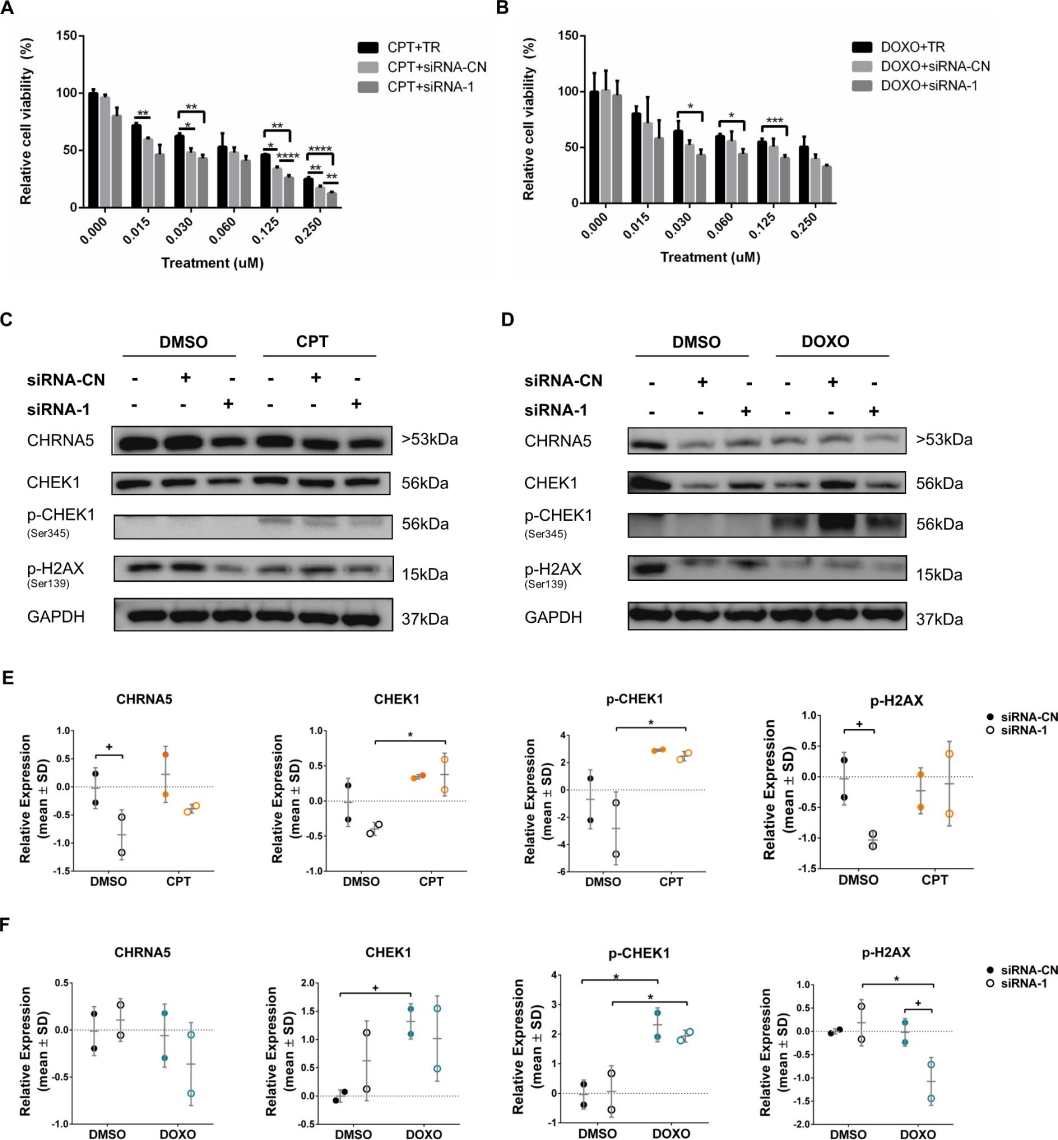
Effects of CHRNA5 RNAi in drug sensitivity in BT-20 cell line. Relative cell viability of siRNA-1 treated BT-20 cells to **A.** CPT and siRNA-1, **B.** DOXO and siRNA-1 treatments at increasing concentrations of CPT and DOXO. **C-F.** Western blots (C, D for siRNA-1 or siRNA-CN in the CPT (0.125μM) and DOXO (0.125μM) groups, respectively) and densitometry results with statistical analysis in CPT (E), DOXO (F) treatment groups with or without siRNA-1, respectively for CHRNA5, CHEK1, pCHEK1, pH2AX. (n = 2 for Western blotting and n = 3 for MTT assays; +: p < 0.1; *: p < 0.05, **: p < 0.01, ***: p < 0.001, ****: p < 0.0001).

**Fig 11:**
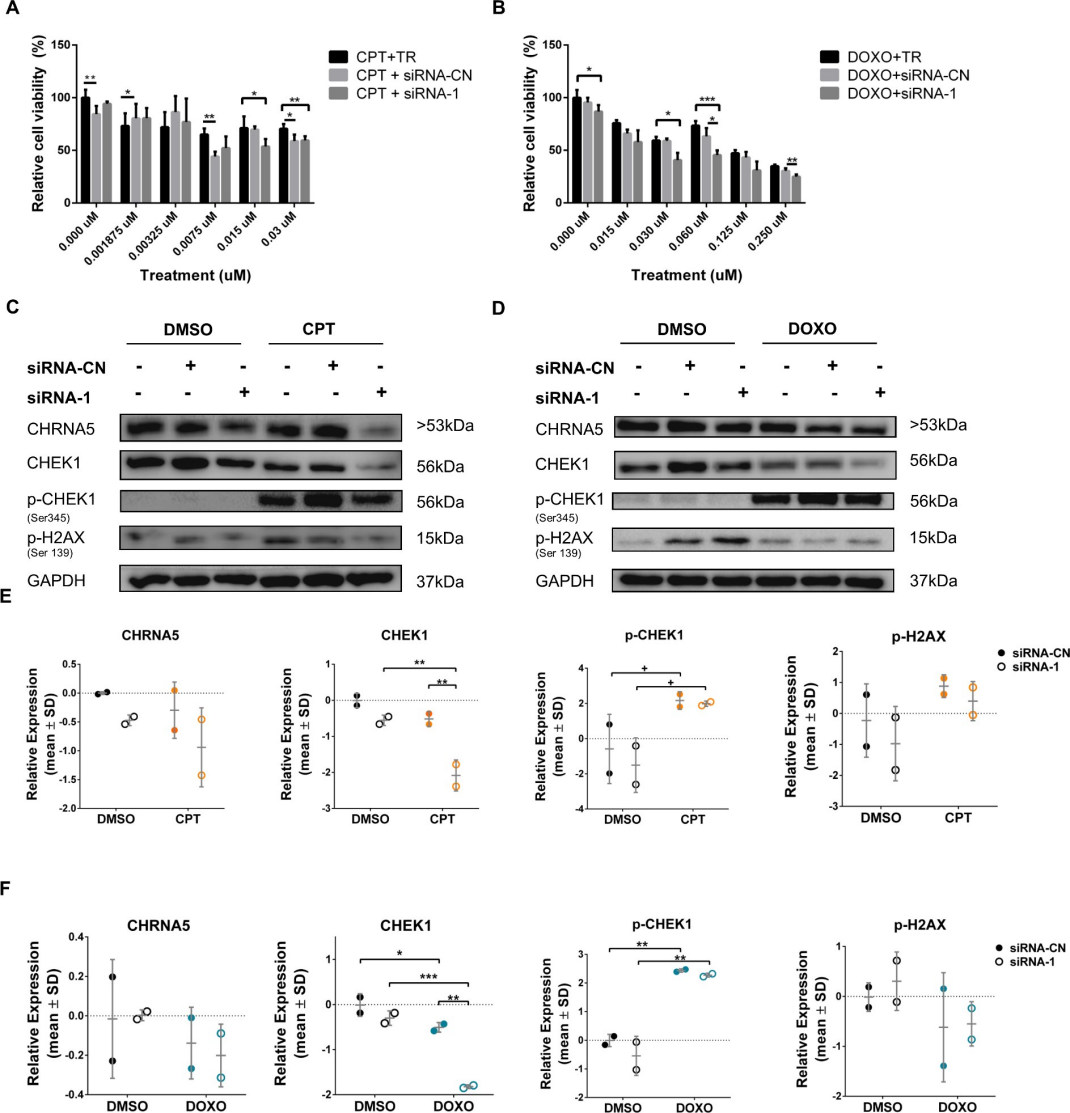
Effects of CHRNA5 RNAi in drug sensitivity in MDA-MB-231 cell line. Relative cell viability of siRNA-1 treated MDA-MB-231 cells to **A.** CPT and siRNA-1, **B.** DOXO and siRNA-1 treatments at increasing concentrations of CPT (0.015μM) and DOXO (0.06μM). **C-F.** Western blots (C, D for siRNA-1 or siRNA-CN in the CPT and DOXO groups, respectively) and densitometry results with statistical analysis in CPT (E), DOXO (F) treatment groups with or without siRNA-1 respectively for CHRNA5, CHEK1, pCHEK1, pH2AX. (n = 2 for Western blotting and n = 3 for MTT assays; +: p < 0.1; *: p < 0.05, **: p < 0.01, ***: p < 0.001, ****: p < 0.0001).

To investigate the effect of siRNA-1 on BT-20 and MDA-MB-231 cell lines, both of which have mutant TP53 status, we tested anti-proliferative effects of CPT/DOXO with or without siRNA-1 using MTT assays (Figs 10A, 10B, 11A and 11B, respectively). The results indicated that the response to siRNA with or without DOXO/CPT could vary between different cell lines. The enhanced anti-proliferative response of BT-20 cells to CPT/DOXO in the presence of CHRNA5 siRNA-1 could only be observed starting from 0.125 μM dose (Fig 10A and 10B) whereas in MCF7 cells CPT/DOXO together with CHRNA5 siRNA-1 decreased cell viability at much lower doses, i.e., <0.015 μM (Fig 8B and 8C). We then demonstrated by Western Blotting that 0.125μM of CPT and DOXO resulted in significantly higher pCHEK1 levels than DMSO control treatment where pCHEK1 expression was low or negligible (Fig 10E and 10F). However, the presence of siRNA-1 could not reverse the induction (Fig 10E and 10F). pH2AX levels were relatively labile in the absence or presence of drugs with or without siRNA (Fig 10E and 10F). In MDA-MB-231 cells, the results were similar with those of BT20 for pCHEK1 levels, i.e., a significant increase in the presence of CPT and DOXO while siRNA-1 treatment had no further effect (Fig 11E and 11F). pH2AX was not significantly affected in MDA-MB-231 cells (Fig 11E and 11F). As opposed to BT-20, total CHEK1 levels decreased upon siRNA treatment in MDA-MB-231 cells in accord with RT-qPCR results (Fig 11E and 11F; S3B Fig).

**Fig 12:**
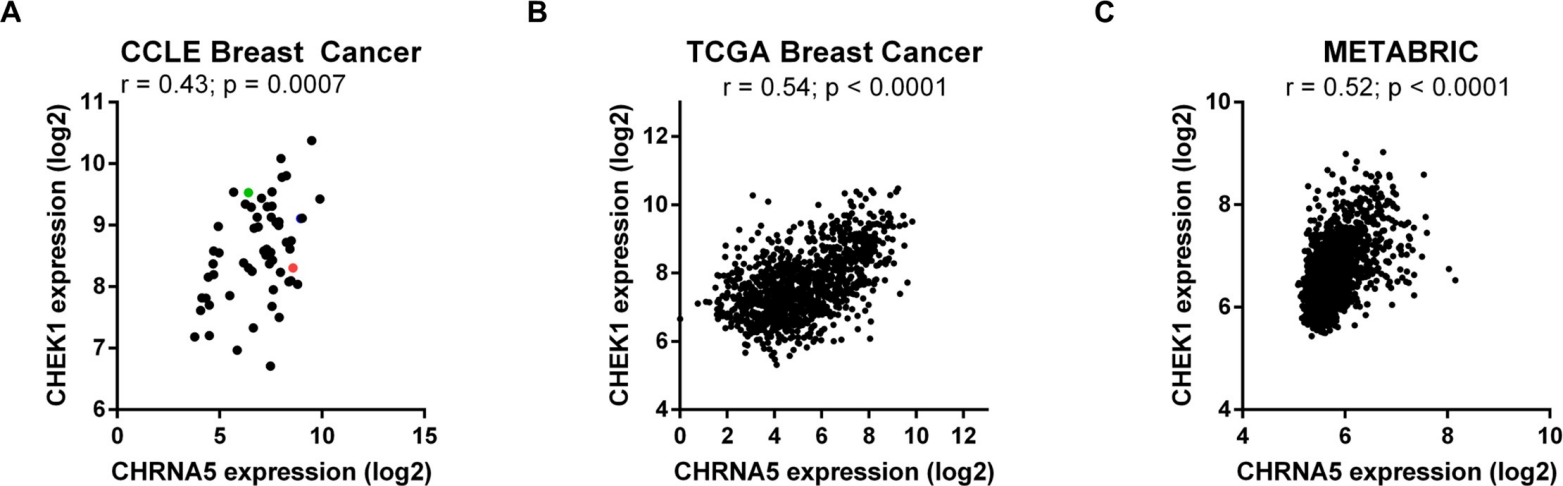
Correlation between CHRNA5 and CHEK1 expression levels. **A-C.** CCLE (A, n = 58), TCGA (B, n = 1100), METABRIC (C, n = 1904). The colors blue, red, and green, respectively, indicate MCF7, BT20, and MDA-MB-231 cell lines in (A).

Since total CHEK1 and pCHEK1 levels were consistently affected by more than one siRNA in our panels, we tested whether CHEK1 mRNA expression was correlated with that of CHRNA5 in CCLE, TCGA, and METABRIC gene expression datasets using cbioportal.org tool. There was a highly significant correlation between CHRNA5 and CHEK1 levels in all three datasets suggesting similar patterns of regulation (Fig 12).

## Discussion

In the present study, we showed using bioinformatics analyses that CHRNA5 was differentially expressed among breast cancer cell lines and tumors with respect to the level of genomic alterations. In addition, the level of CHRNA5 expression differed between TP53 wild type and mutant tumors while at the same time was highly correlated with that of CHEK1 expression, whose inhibition known to enhance chemosensitivity [23]. We also observed MCF7 breast cancer cell line expressed multiple CHRNA5 isoforms, all of which could be significantly downregulated by using RNAi molecules. Similarly, the expression level of CHRNA5 was successfully downregulated in TP53 mutant BT-20 and MDA-MB-231 cells. Our study demonstrated a significant antimitotic effect of CHRNA5 RNAi application in breast cancer cell lines, complementing the results of existing RNAi phenotypic screen findings in other cell types [63–65]. By transcriptomic profiling of MCF7 cells exposed to CHRNA5 RNAi, we identified significant changes in multiple cancer related pathways some of which were previously shown for A549 lung cancer cell line [47]. We used comparative transcriptomics to test correlation of CHRNA5 RNAi expression signature in MCF7 cells with the expression profiles from CCLE breast cancer cell lines, and from nutlin-3a induced as well as from topoisomerase inhibited MCF7 cells. This type of comparative transcriptomics approach has proven useful in generalizing findings thus helped us identify common and unique features of the CHRNA5 RNAi treatment. We validated multiple aspects of the CHRNA5 RNAi application, i.e., cell cycle inhibition and G1/S arrest, apoptosis, and reduced DDR using different methodologies. We proposed that the effects of CHRNA5 RNAi might include secondary transcriptomic responses in addition to the primary ones. The observation that 120h of exposure to siRNA-1 treatment exhibited similar but stronger responses supported the involvement of downstream effectors in response to CHRNA5 depletion. We finally demonstrated an enhancement in the chemosensitivity of breast cancer cells to CPT and DOXO in the presence of CHRNA5 RNAi yet in dose- and cell-specific manners. Consistent CHEK1 inhibition (both total and phosphorylated) was among the most significant findings observed with all three siRNAs in MCF7 cells. In addition, the correlation of mRNA expressions of CHRNA5 and CHEK1 among breast cancer cell lines and tumors was highly significant. Lower levels of total and phosphorylated CHEK1 could potentially lead to interruption in DDR [66]. Recently CHEK1 inhibition has gained enormous attention and new CHEK1 inhibitors are being developed [28]. Our findings reveal that CHRNA5 RNAi could act as a candidate CHEK1 inhibitor leading to enhanced drug sensitivity in breast cancer cells and warrants further studies in different cell lines and cancers.

Previously CHRNA3/CHRNA5/CHRNB4 locus and nearby gene PSMA4 have been associated with increased risk of lung cancer [67]. It is known that adjacent and non-adjacent neighboring genes can exhibit highly correlated expression profiles at mRNA level due to their shared genomic regulation as well as functional similarity [68, 69]. In support of this, we showed that several chromosomal neighbors of CHRNA5 exhibited high correlation of expression also in breast cancer; interestingly, many of these were also involved in cell cycle and proliferation (S2 Table).

In accord with previously shown role of CHRNA5 in cell adhesion and motility[15], we identified significant changes in the expressions of several cell junction and motility genes. Among these, MAP1B, whose expression significantly upregulated by CHRNA5 RNAi, functions in axonal elongation of neurons [70], microtubule regulation of MDA-MB-231 [71] as well as cell differentiation in the PC12 cell line [72]. CLDN1, is associated with apoptosis, EMT, and prognosis in breast cancer [73–78]. The expressions of MAP1B and CLDN1 have also been upregulated in BT-20 cells, thus regardless of TP53 status. Therefore, the effects of CHRNA5 RNAi on cell-cell junctions and differentiation are promising and can be further studied for their roles in tumor progression.

Our experimental studies demonstrate antiproliferative effects of CHRNA5 depletion in breast cancer cell lines using multiple approaches, i.e., RT-qPCR, Western blotting, and comparative transcriptomics analyses. As a result, we found reduction in pRB protein levels, which can restrict progress from G1 to S phase [79]. A significant increase in the mRNA expression of p21/CDKN1A, an inhibitor of RB [80], and reduction in the expression of CCNE2, which activates CDK2 for phosphorylation of RB [61, 81], support the cell cycle arrest phenotype. However, the differential response of CCND1 mRNA levels to multiple siRNA molecules targeting CHRNA5 requires further study. Our results hence suggest that CHRNA5 depletion might be associated with cell cycle arrest during G1 phase through modulation of RB, increased CDKN1A and reduced CCNE2 levels in MCF7 cells.

Decreased pRB levels can also result in dissociation of RB from BAX, which is able to stimulate apoptosis [82]. Cell cycle analysis of sub-G1 population in MCF7 cells has supported the presence of cell death in response to CHRNA5 depletion. Upregulated FAS at the mRNA and protein levels also points to a possible FAS-induced apoptotic pathway. This could be further enhanced with the intrinsic pathway reflected by the increased levels of BAX/BCL2 ratio (both at mRNA and protein levels) [83] although only siRNA-1 resulted in increased levels of cleaved CASP7 [84, 85]. mRNA and protein levels of BAX and BCL2 genes correlate well and thus an increased BAX/BCL2 ratio could mark a pro-apoptotic response [84–86].

In this study we have also shown that CHRNA5 RNAi might result in reduced DNA damage response (DDR) based on TCGA and METABRIC tumor data, and GSEA and DAVID functional analyses as well as quantification of total and phosphorylated CHEK1 protein levels. The observed positive correlations between CHRNA5 and CHEK1 mRNA expression levels in CCLE, TCGA and METABRIC breast cancer datasets are supportive of CHRNA5 RNAi findings in MCF7. However, pH2AX protein levels were relatively labile and future studies should focus on comparison with total H2AX levels and/or changes in cellular localization. Our results suggest that CHRNA5 RNAi could act as a potential CHEK1 inhibitor, which is known to reduce DDR and help sensitize cells to topoisomerase inhibitors [87].

TP53 is a gatekeeper protein in G1/S checkpoint arrest preventing cells to undergo early entry to S phase and mitosis in response to ATR/ATM activity in DNA damage [88]. In the drug sensitivity assays we used TP53 wild type luminal MCF7 cell line, one of the most tested *in vitro* models for understanding the mechanisms behind chemoresistance [89] as well as TP53 mutant cell lines, BT-20 and MDA-MB-231. Interestingly, we found that pCHEK1 was significantly induced by CPT and DOXO in TP53 mutant cell lines suggesting presence of an intact DDR mechnanism [90] while in MCF7 cells it was significantly decreased in response to CPT and DOXO. In addition, CHRNA5 RNAi treatment increased sensitivity even at very low doses of DOXO and CPT in MCF7 cells. This sensitization effect might depend on highly reduced CHEK1 mRNA expression levels as well as CHEK1 activity, measured by pCHEK1 (S345) levels upon siRNA exposure [20, 21]. Overall, our findings imply that CHRNA5 RNAi treatment can sensitize the TP53 wild type MCF7 cells possibly through reduced CHEK1 and activated TP53 pathways; on the other hand, in the TP53 mutant cell lines the sensitizing effects could be less pronounced potentially due to induction of pCHEK1 in the presence of topoisomerase inhibitors.

Our findings implicate depletion of CHRNA5 with significant alterations in proteins involved in apoptosis and DDR. Application of a multivariate approach such as DFA for analysis of Western blotting results has helped us show that effects of different topoisomerases as well siRNA molecules could be discriminated better based on linear combinations of these proteins. This analysis also showed that the effects of siRNA-1 and -2 were more similar to each other than that of siRNA-3 in the DMSO treatment. In addition, CPT and DOXO treatment effects on the studied proteins could be discriminated from each other. Our results also signify caution such that cell lines may not be equally sensitive to CHRNA5 depletion. This could be due to differences in TP53 status as well as mechanisms other than modulation of DDR or apoptosis, such as hormone receptor expression and activity.

In this study, we showed that depletion of CHRNA5 in MCF7 cells resulted in decreased pRB, CHEK1 and pCHEK1 levels and increased BAX/BCL2 ratio as well as enhanced sensitivity to TOPO inhibitors at certain concentrations. In addition, TP53 mutant cell lines exhibited differences in response to topoisomerase inhibitors [91, 92] that might counteract the effects of CHRNA5 depletion on CHEK1 and hence pCHEK1 levels. We propose that these observed alterations potentially affecting cell fate and survival could partly be achieved through changes in the cholinergic receptor composition by CHRNA5 RNAi in breast cancer cells. Previous studies have shown that incorporation or exclusion of CHRNA5 from the cholinergic receptor’s pentameric structure can modulate intracellular calcium levels [93, 94]; future studies in this direction could provide novel insights about the mechanisms of action by which CHRNA5 depletion works in breast tumors. It is also crucial that future studies should demonstrate reversal of phenotype by overexpression studies. In addition, the therapeutic response of breast tumors exhibiting variable CHRNA5 levels to TOPO inhibitors as well as effects of CHRNA5 RNAi on isogenic cell line models with differing ER and TP53 status should be studied.

## Supporting information

S1 Table Primer pairs used in this study for gene expression analysis by RT-qPCR. (PDF)

S2 Table KEGG annotation of shared CHRNA5 siRNA-1 and CCLE expression profile in unfiltered/filtered (expression > 5; described in results section) correlation analysis (p values < 0.05). NA represents term that was not found significant.
(PDF)

S3 Table Chromosomal neighbors correlated with CHRNA5 expression in siRNA-1 and CCLE expression profiles. (PDF)

S4 Table KEGG pathway analysis of CHRNA5 siRNA profiles in common between MCF7 and A549 cell lines. (PDF)

S5 Table Fold changes of DDR genes in Fig 7 in microarray data and their correlation scores in TCGA and METABRIC. (PDF)

S1 Fig Validation studies of CHRNA5 RNAi model at 120h exposure. **A.** RT-qPCR analysis of CHRNA5 depletion for *CHRNA5_v1* isoform. **B.** Western Blotting of siRNA-1 and siRNA-CN (10nM) treated MCF7 cells. **C.** RT-qPCR analysis of selected genes upon 10nM siRNA-1 treatment for 120h in comparison with 72h values from Fig 4A in MCF7 cells (n = 1 for siRNA-CN and n = 2 for siRNA-1). Expression of SDHA gene is used as reference. **D.** Western blot analysis of pRB, total CASP7, cleaved CASP7, BCL2, BAX, total CHEK1, pCHEK1, pH2AX proteins from siRNA-1 and siRNA-CN treated groups (n = 2 per group). **E.** Densitometry analysis and statistical comparisons. Student’s t-test was applied. (*: p < 0.05, **: p < 0.01, #: p < 0.001).
(TIF)

S2 Fig Validations for RNAi molecules in MCF7, BT-20 and MDA-MB-231 cells. **A.** CHRNA5 levels in siRNA-1 treated BT20 and MDA-MB-231 cell line (n = 2 per group). **B.** Relative cell viability of MCF7 cells upon siRNA-1-3 exposure (n = 3 per group). **C-D.** Relative cell viability of BT20 (C) MDA-MB-231 upon siRNA-1 exposure (D) (n = 3 per group). (*: p < 0.05, **: p < 0.01, ***: p < 0.001, ****: p < 0.0001).
(TIF)

S3 Fig Validations for apoptosis, cyclin and DDR related expression in breast cancer cell lines. **A-B.** One-Way ANOVA of densitometry measurements of cleaved CASP7/total CASP7 ratio (A) and pH2AX (B) in MCF7 cells. siRNA-CN (10nM) and siRNA-CN (50nM) were used as control groups for siRNA-1, and siRNA-2-3, respectively. (n = 2 per group for siRNA-CN (10nM) and siRNA-1; n = 3 per group for siRNA-CN (50nM) and siRNA-2 and -3). **C.** FAS and BID protein levels upon siRNA-1 treatment for 72h (left) and 120h (right) in MCF7 cells and densitometry analysis with student’s t-test. **D.** RT-qPCR analysis of selected genes after 10nM siRNA-1 treatment for 72h in MDA-MB-231 and BT-20 cells in comparison with results from MCF7 shown Fig 6I. **E.** BAX/BCL2 ratio in BT-20 and MDA-MB-231 in comparison with MCF7 cells (data from Fig 6J), after siRNA-1 exposure (*: p < 0.05, **: p < 0.01).
(TIF)

S4 Fig Expression analysis of CHRNA5 expression with respect to TP53 status. **A-B** METABRIC (A) and TCGA(B) datasets for TP53 mutant and wild type patients.
(TIF)

S5 Fig Preliminary analysis of relative cell viability in CHRNA5 siRNA-1 treated cells in response to topoisomerase inhibitors Camptothecin (CPT) and Doxorubicin (DOXO). **A-B.** Relative cell viability of 72h exposure of CPT (0-2uM) (A) or DOXO (0-2uM) (B) and 10nM siRNA-1 treated MCF7 cells, or in combination with the corresponding siRNA-CN controls. Treatments having the same drug and DMSO concentrations were shown on the x-axis as groups; Labels: drug alone (a), drug+siRNA-CN (b), and drug+siRNA-1(c). Letters on top of the siRNA-CN (b) or siRNA-1 exposed (c) treatments are labels indicating the treatment identity (a, b, or c, as defined above) significantly different based on Tukey HSD corrected One-Way ANOVA results (n = 3 per group; p adj. <0.05).
(TIF)

S6 Fig Densitometry measurements. **A-B.** Two-Way ANOVA of densitometry measurements of cleaved CASP7/total CASP7 (A) and pH2AX (B) in DMSO, CPT, and DOXO groups.
(TIF)
