# Supplementary material for: Cholinergic Receptor Nicotinic Alpha 5 (CHRNA5) RNAi is associated with cell cycle inhibition, apoptosis, DNA damage response and drug sensitivity in breast cancer
Source: PLoS One. 2018 Dec 13;13(12):e0208982. doi: 10.1371/journal.pone.0208982 (PMC6292578; doi:10.1371/journal.pone.0208982)
Supplement: S1 Table — (PDF) [file pone.0208982.s001.pdf]

S1 Table. Primer pairs used in this study for gene expression analysis by RT-qPCR

Name	Primer sequence (5'-3')	Size of amplicon (bp)	Amplification efficiency
CHRNA5_v1	F: AGATGGAACCCTGATGACTATGGT	104	1.87
	R: AAACGTCCATCTGCATTATCAAAC		
CHRNA5_v2	F: GGAAACTGAGAGTGGTAGTGGA	122	1.95
	R: CTTCAACAACCTCACGGACA		
CHRNA5_v3	F: CATCAGGTGTTGAAGATTGGAAAT	101	1.92
	R: AAAAAGCCCAAGAGATCCAACAAT		
CHRNA5_iso2*	F: TGGAGAATGGGAGATTGTGAGTGCA	78	1.97
	R: CCAATCTTCAACAACCAGCAACAGC		
CHRNA5_iso3*	F: TGGAGAATGGGAGATTGTGAGTGCA	84	1.96
	R: CCAATCTTCAACAACGGATACCAGC		
SDHA	F: TGGGAACAAGAGGGCATCTG	86	2.01
	R: CCACCACTGCATCAAATTCATG		
MAP1B	F: GTTGGAAGGAAAGGCTCAGT	110	1.81
	R: CTTGCTGTTTCTCATGGGTC		
CLDN1	F: CTGTCATTGGGGGTGCGATA	118	1.88
	R: CTGGCATTGACTGGGGTCAT		
WDHD1	F: AGCAGCCAAGGACGAGTAAA	192	1.95
	R: CTTCGGCTTTGGAATCAGAG		
ANLN	F: TAAAGCAGGTGATTGTTCGG	180	1.97
	R: GTTCTTCATCAACACAGCAG		
BIRC5	F: GTTGCGCTTTCCTTTCTGTC	141	1.92
	R: TCTCCGCAGTTTCCTCAAAT		
CDKN1A	F: GTCACTGTCTTGTACCCTTGTG	129	1.82
	R: CGGCGTTTGGAGTGGTAGAA		
GPNMB	F: TGCTGACTGTGAGACGAACC	204	1.92
	R: ACACCAAGAGGGAGATCACAG		
GJA1	F: TCTGAGTGCCTGAACTTGCC	171	2
	R:CCCTCCAGCAGTTGAGTAGG		
GADD45A	F:TCTCGGCTGGAGAGCAGAAGAC	121	1.96
	R:AGCTTGGCCGCTTCGTACAC		
BAX	F:GGGTTGTCGCCCTTTTCTAC	198	2.09
	R:CTGGAGACAGGGACATCAGT		
BCL2	F:TGAACTGGGGGAGGATTGTG	183	2.04
	R:CGTACAGTTCCACAAAGGCA		
FAS	F:AATAAACTGCACCCGGACCC	192	2.06
	R:AGAAGACAAAGCCACCCCAA		
CCND1	F:CTGCGAAGTGGAAACCATCC	199	1.97
	R:GCACTTCTGTTCCTCGCAGA		
CCNE2	F:GTAGCTGGTCTGGCGAGGTTT	83	2.08
	R:GGGCTGCTGCTTAGCTTGTAA		
CHEK1	F:TGGTCACAGGAGAGAAGGCA	151	1.72
	R:CAGATAAACCACCCCTGCCA		
