# Supplementary material for: Cholinergic Receptor Nicotinic Alpha 5 (CHRNA5) RNAi is associated with cell cycle inhibition, apoptosis, DNA damage response and drug sensitivity in breast cancer
Source: PLoS One. 2018 Dec 13;13(12):e0208982. doi: 10.1371/journal.pone.0208982 (PMC6292578; doi:10.1371/journal.pone.0208982)
Supplement: S2 Table — NA represents term that was not found significant. (PDF) [file pone.0208982.s002.pdf]

S2 Table. KEGG annotation of shared CHRNA5 siRNA-1 and CCLE expression profile in unfiltered/filtered (expression > 5; described in results section) correlation analysis (p values < 0.05). NA represents term that was not found significant.

**CCLE correlations based on unfiltered data / filtered data (expression>5)**			
**Upregulated by siRNA-1 and positively correlated with CHRNA5 in CCLE breast data**			
Term	Count	PValue	Benjamini
hsa04530:Tight junction	11/6	1.59E-04 / 3.09E-02	1.84E-02 / 7.85E-01
hsa04012:ErbB signaling pathway	6 / NA	2.18E-02 / NA	7.24E-01/ NA
hsa04514:Cell adhesion molecules (CAMs)	7 / NA	3.46E-02 / NA	7.47 E-01 / NA
hsa04660:T cell receptor signaling pathway	6 / NA	4.87E-02/ NA	7.68E-01 / NA
hsa03010:Ribosome	NA / 5	NA / 2.71E-02	0 / 9.32E-01
**Downregulated by siRNA-1 and negative correlated with CHRNA5 in CCLE breast data**			
hsa04144:Endocytosis	7 / 7	2.73E-02 / 2.37E-02	8.94E-01 / 8.27E-01
hsa04810:Regulation of actin cytoskeleton	NA / 7	NA / 4.57E-02	NA / 8.19E-01
**Upregulated by siRNA-1 and negatively correlated with CHRNA5 in CCLE breast data**			
hsa05219:Bladder cancer	5 / 5	4.61E-03 / 5.02E-03	4.09E-01 / 2.34E-01

hsa00520:Amino sugar and nucleotide sugar metabolism	5 / 4	5.45E-03 / 3.65E-02	2.68E-01 / 3.89E-01
hsa04310:Wnt signaling pathway	8 / 9	1.11E-02 / 3.49E-03	3.47E-01 / 3.10E-01
hsa04510:Focal adhesion	9 / 9	1.61E-02 / 1.84E-02	3.70E-01 / 3.88E-01
hsa00500:Starch and sucrose metabolism	4 / NA	3.05E-02 / NA	5.06E-01 / NA
hsa04142:Lysosome	6 / NA	4.15E-02 / NA	5.53E-01 / NA
hsa04350:TGF-beta signaling pathway	NA / 6	NA / 1.47E-02	NA / 4.07E-01
hsa05200:Pathways in cancer	NA / 12	NA / 1.97E-02	NA / 3.45E-01
hsa04115:p53 signaling pathway	NA / 5	NA / 2.63E-02	NA / 3.76E-01
hsa04144:Endocytosis	NA / 8	NA / 3.33E-02	NA / 4.01E-01
hsa00520:Amino sugar and nucleotide sugar metabolism	NA / 4	NA / 3.65E-02	NA / 3.89E-01
**Downregulated by siRNA-1 and positively correlated with CHRNA5 in CCLE breast data**			
hsa03030:DNA replication	24 / 24	1.33E-22 / 2.48E-24	2.0E-20 / 3.25E-22
hsa04110:Cell cycle	34 / 31	6.04E-17 / 2.63E-16	8.44E-15 / 1.45E-14
hsa03430:Mismatch repair	13 / 13	7.56E-11 / 1.03E-11	3.81E-09 / 3.37E-10
hsa03040:Spliceosome	27 / 27	7.59E-11 / 1.52E-12	2.87E-09 / 6.65E-11
hsa03410:Base excision repair	14 / 14	2.32E-09 / 2.86E-10	7.01E-08 / 7.49E-09
hsa00240:Pyrimidine metabolism	21 / 20	9.18E-09 / 3.27E-09	2.31E-07 / 7.14E-08
hsa03420:Nucleotide excision repair	13 / 13	4.87E-07 / 7.68E-08	1.05E-05 / 1.44E-06

hsa03440:Homologous recombination	10 / 10	3.04E-06 / 7.39E-07	5.73E-05 / 1.21E-05
hsa00230:Purine metabolism	22 / 19	7.35E-06 / 2.89E-05	1.23E-04 / 3.79E-04
hsa03018:RNA degradation	12 / 12	5.31E-05 / 1.10E-05	8.01E-04 / 1.60E-04
hsa04114:Oocyte meiosis	16 / 15	1.67E-04/9.79E-05	2.29E-03/ 1.16E-03
hsa04914:Progesterone-mediated oocyte maturation	12 / 10	2.11E-03/ 6.69E-03	2.62E-02/ 7.06E-02
hsa04115:p53 signaling pathway	9 / 8	1.36E-02/ 1.75E-02	1.47E-01 / 1.63E-01
hsa00900:Terpenoid backbone biosynthesis	4 / 4	3.07E-02 / 1.97E-02	2.85E-01 / 1.70E-01
hsa00670:One carbon pool by folate	4 / 4	3.65E-02 / 2.36E-02	3.12E-01 / 1.88E-01
