# Supplementary material for: Cholinergic Receptor Nicotinic Alpha 5 (CHRNA5) RNAi is associated with cell cycle inhibition, apoptosis, DNA damage response and drug sensitivity in breast cancer
Source: PLoS One. 2018 Dec 13;13(12):e0208982. doi: 10.1371/journal.pone.0208982 (PMC6292578; doi:10.1371/journal.pone.0208982)
Supplement: S3 Table — (PDF) [file pone.0208982.s003.pdf]

S3 Table. Chromosomal neighbors correlated with CHRNA5 expression in siRNA-1 and CCLE expression profiles.

ID	Gene Name	Species
203755_AT	BUB1 mitotic checkpoint serine/threonine kinase B(BUB1B)	Homo sapiens
205733_AT	Bloom syndrome RecQ like helicase(BLM)	Homo sapiens
213007_AT	Fanconi anemia complementation group I(FANCI)	Homo sapiens
1558750_A_AT	OTU deubiquitinase 7A pseudogene(LOC100288637)	Homo sapiens
213599_AT	Opa interacting protein 5(OIP5)	Homo sapiens
228252_AT	PIF1 5'-to-3' DNA helicase(PIF1)	Homo sapiens
219258_AT	TIMELESS interacting protein(TIPIN)	Homo sapiens
230021_AT	TOPBP1 interacting checkpoint and replication regulator(TICRR)	Homo sapiens
239680_AT	WD repeat domain 76(WDR76)	Homo sapiens
239413_AT	centrosomal protein 152(CEP152)	Homo sapiens
206533_AT	cholinergic receptor nicotinic alpha 5 subunit(CHRNA5)	Homo sapiens
202705_AT	cyclin B2(CCNB2)	Homo sapiens
208955_AT	deoxyuridine triphosphatase(DUT)	Homo sapiens
224779_S_AT	family with sequence similarity 96 member A(FAM96A)	Homo sapiens
202070_S_AT	isocitrate dehydrogenase 3 (NAD(+)) alpha(IDH3A)	Homo sapiens
204709_S_AT	kinesin family member 23(KIF23)	Homo sapiens
225300_AT	kinetochore localized astrin/SPAG5 binding protein(KNSTRN)	Homo sapiens
218039_AT	nucleolar and spindle associated protein 1(NUSAP1)	Homo sapiens
218009_S_AT	protein regulator of cytokinesis 1(PRC1)	Homo sapiens
205768_S_AT	solute carrier family 27 member 2(SLC27A2)	Homo sapiens
222606_AT	zwilch kinetochore protein(ZWILCH)	Homo sapiens
