# Supplementary material for: Cholinergic Receptor Nicotinic Alpha 5 (CHRNA5) RNAi is associated with cell cycle inhibition, apoptosis, DNA damage response and drug sensitivity in breast cancer
Source: PLoS One. 2018 Dec 13;13(12):e0208982. doi: 10.1371/journal.pone.0208982 (PMC6292578; doi:10.1371/journal.pone.0208982)
Supplement: S4 Table — (PDF) [file pone.0208982.s004.pdf]

S4 Table : Top 5 KEGG pathways (having BH<0.05) of shared CRHNA5 depleted transcriptome in MCF7 and A549 cell line.

Term	Count	PValue	Benjamini
**Upregulated in both MCF7 and A549**			
Term	Count	PValue	Benjamini
hsa03060:Protein export	10	8.22E-06	2.16E-03
hsa04141:Protein processing in endoplasm	25	3.01E-04	3.89E-02
**Downregulated in both MCF7 and A549**			
Term	Count	PValue	Benjamini
hsa03030:DNA replication	28	1.51E-21	4.07E-19
hsa04110:Cell cycle	51	2.03E-21	2.73E-19
hsa03040:Spliceosome	49	3.03E-18	2.73E-16
hsa03460:Fanconi anemia pathway	26	3.78E-13	2.55E-11
hsa03440:Homologous recombination	18	2.58E-11	1.39E-09
**Upregulated in MCF7 and downregulated in A549**			
Term	Count	PValue	Benjamini
hsa05203:Viral carcinogenesis	30	6.13E-07	1.57E-04
hsa05161:Hepatitis B	24	1.25E-06	1.60E-04
hsa05160:Hepatitis C	21	1.43E-05	1.22E-03
hsa05142:Chagas disease (American trypan	18	2.08E-05	1.33E-03
hsa04917:Prolactin signaling pathway	14	5.86E-05	2.99E-03
**Downregulated in MCF7 and Upregulated in A549**			
Term	Count	PValue	Benjamini
hsa04931:Insulin resistance	18	2.49E-05	6.41E-03
