# Supplementary material for: Cholinergic Receptor Nicotinic Alpha 5 (CHRNA5) RNAi is associated with cell cycle inhibition, apoptosis, DNA damage response and drug sensitivity in breast cancer
Source: PLoS One. 2018 Dec 13;13(12):e0208982. doi: 10.1371/journal.pone.0208982 (PMC6292578; doi:10.1371/journal.pone.0208982)
Supplement: S5 Table — (PDF) [file pone.0208982.s005.pdf]

S5 Table. Fold changes of DDR genes in Fig 7 in microarray data and their correlation scores in TCGA and METABRIC.

		TCGA			Metabric		
**Gene Symbol**	**Gene ID**	**r score**	**log FC**	**p Value**	**r score**	**log FC**	**p Value**
APEX1	ENSG00000100823	0.0587451	0.103532064	0.254852828	0.060826831	0.103532064	0.254852828
ATM	ENSG00000149311	-0.013366638	0.052742369	0.550580148			
ATR	ENSG00000175054	0.184065596	-0.297649961	0.006215572	0.137416627	-0.297649961	0.006215572
BRCA1	ENSG00000012048	0.057960583	-1.227567621	1.68313E-05	0.034661631	-1.227567621	1.68313E-05
BRCA2	ENSG00000139618	0.324952261	-0.772322573	5.68919E-05	-0.024774271	-0.772322573	5.68919E-05
CCNH	ENSG00000134480	-0.325734021	-0.069545803	0.400412521	-0.154056628	-0.069545803	0.400412521
CDK7	ENSG00000134058	-0.161804682	0.095047495	0.313264612	-0.141782828	0.095047495	0.313264612
CHEK1	ENSG00000149554	0.51039426	-1.045513363	7.36251E-06	0.516652113	-1.045513363	7.36251E-06
CHEK2	ENSG00000183765	0.336496349	-0.655084814	0.000293493	0.358327403	-0.655084814	0.000293493
DDB1	ENSG00000167986	0.092872696	-0.053981657	0.506830608	0.189071813	-0.053981657	0.506830608
DUT	ENSG00000128951	0.136583952	-0.704490198	5.94182E-05	0.111335597	-0.704490198	5.94182E-05
ERCC1	ENSG00000012061	-0.22344735	0.311090712	0.022085355	0.014357655	0.311090712	0.022085355
ERCC2	ENSG00000104884	-0.111993498	0.047220623	0.666228489	-0.059368896	0.047220623	0.666228489
ERCC6	ENSG00000225830	0.038300306	0.317522737	0.036927959			
ERCC8	ENSG00000049167	0.028015231	-0.296806149	0.007204485			
EXO1	ENSG00000174371	0.393951091	-1.055779646	0.000274244	0.41177178	-1.055779646	0.000274244
FANCC	ENSG00000158169	0.321429004	-0.505603164	0.000568918	0.085861533	-0.505603164	0.000568918
FANCD2	ENSG00000144554	0.361227897	-1.132880491	3.64968E-05	0.316458768	-1.132880491	3.64968E-05
FANCF	ENSG00000183161	-0.018267684	0.64173401	0.000580434			
FANCG	ENSG00000221829	0.23949625	-1.027954566	1.50371E-05	0.146611331	-1.027954566	1.50371E-05
H2AFX	ENSG00000188486	0.244992331	-0.502951538	0.00178265	0.305201626	-0.502951538	0.00178265
HLTF	ENSG00000071794	0.152801809	-0.558600892	0.0004028	0.097146429	-0.558600892	0.0004028
LIG4	ENSG00000174405	0.065247321	0.502385023	0.001454301	0.03525646	0.502385023	0.001454301
MAD2L2	ENSG00000116670	0.214613023	-0.737974807	0.001020095	0.250013361	-0.737974807	0.001020095
MBD4	ENSG00000129071	0.166396715	-0.238015265	0.019475793			
MGMT	ENSG00000170430	-0.253473033	-0.187466946	0.114461118	-0.264063372	-0.187466946	0.114461118
MLH1	ENSG00000076242	-0.071989668	-1.345076533	4.97846E-06	-0.051771634	-1.345076533	4.97846E-06
MLH3	ENSG00000119684	-0.184014506	0.239812887	0.038198445	-0.114373148	0.239812887	0.038198445
MRE11A	ENSG00000020922	0.222839897	-0.786664368	3.05792E-05	0.011403411	-0.786664368	3.05792E-05
MSH2	ENSG00000095002	0.485502405	-0.611817684	0.0001271	0.192959904	-0.611817684	0.0001271
MSH3	ENSG00000113318	-0.185073183	-0.332032958	0.065029003	-0.222895377	-0.332032958	0.065029003
MSH6	ENSG00000116062	0.499587279	-0.716899791	5.55811E-05	0.300568505	-0.716899791	5.55811E-05
MUS81	ENSG00000172732	0.013019756	0.116856228	0.185976931	-0.003748789	0.116856228	0.185976931
NBN	ENSG00000104320	0.166544095	-0.208336054	0.033138482	0.056687584	-0.208336054	0.033138482
NEIL3	ENSG00000109674	0.355468866	-1.408404221	2.76285E-06	0.352807319	-1.408404221	2.76285E-06
OGG1	ENSG00000114026	-0.062858865	-0.001251532	0.987120824	0.107214495	-0.001251532	0.987120824
PARP1	ENSG00000143799	0.163824497	-0.428683392	0.001434718			
PCNA	ENSG00000132646	0.357798865	-0.700492314	6.43262E-05	0.199545935	-0.700492314	6.43262E-05
POLD1	ENSG00000062822	0.201552847	-0.871572341	6.11758E-05	0.22316662	-0.871572341	6.11758E-05
PRKDC	ENSG00000253729	0.332457839	-0.049474885	0.600803014			
RAD23B	ENSG00000119318	0.084665772	0.16777282	0.085779871	0.071937243	0.16777282	0.085779871
RAD50	ENSG00000113522	-0.164812324	0.427992556	0.002329211			
RAD51	ENSG00000051180	0.386886535	-0.495832042	0.00142255	0.331802787	-0.495832042	0.00142255
RAD52	ENSG00000002016	-0.081290859	-0.063461247	0.607844051			
RPA2	ENSG00000117748	0.064736852	-0.642001589	0.000144523	-0.036720914	-0.642001589	0.000144523
RRM2B	ENSG00000048392	0.016976032	1.000011155	0.000151078	0.05413382	1.000011155	0.000151078
TP53	ENSG00000141510	-0.020551438	-0.246480808	0.024849998			

TP53BP1	ENSG00000067369	-0.133135905	-0.18472387	0.048027543	-0.156315577	-0.18472387	0.048027543
WRN	ENSG00000165392	0.108390358	-0.218201635	0.033282267	0.009233785	-0.218201635	0.033282267
XPA	ENSG00000136936	-0.377699998	0.336911743	0.013571621	-0.079811444	0.336911743	0.013571621
XPC	ENSG00000154767	-0.329075827	0.875034147	1.95921E-05	-0.293896093	0.875034147	1.95921E-05
XRCC1	ENSG00000073050	-0.168781058	-0.081621975	0.335502339	-0.13020509	-0.081621975	0.335502339
XRCC3	ENSG00000126215	0.114802393	-0.413969088	0.005088717	0.164741377	-0.413969088	0.005088717
XRCC4	ENSG00000152422	0.020962873	-0.600134983	0.000223004			
XRCC5	ENSG00000079246	0.178396517	-0.364894565	0.005471218	0.108148952	-0.364894565	0.005471218
XRCC6	ENSG00000196419	0.27434471	-0.479711109	0.000980726	0.188782783	-0.479711109	0.000980726
