# Supplementary figures and images for: Cholinergic Receptor Nicotinic Alpha 5 (CHRNA5) RNAi is associated with cell cycle inhibition, apoptosis, DNA damage response and drug sensitivity in breast cancer
Source: PLoS One. 2018 Dec 13;13(12):e0208982. doi: 10.1371/journal.pone.0208982 (PMC6292578; doi:10.1371/journal.pone.0208982)

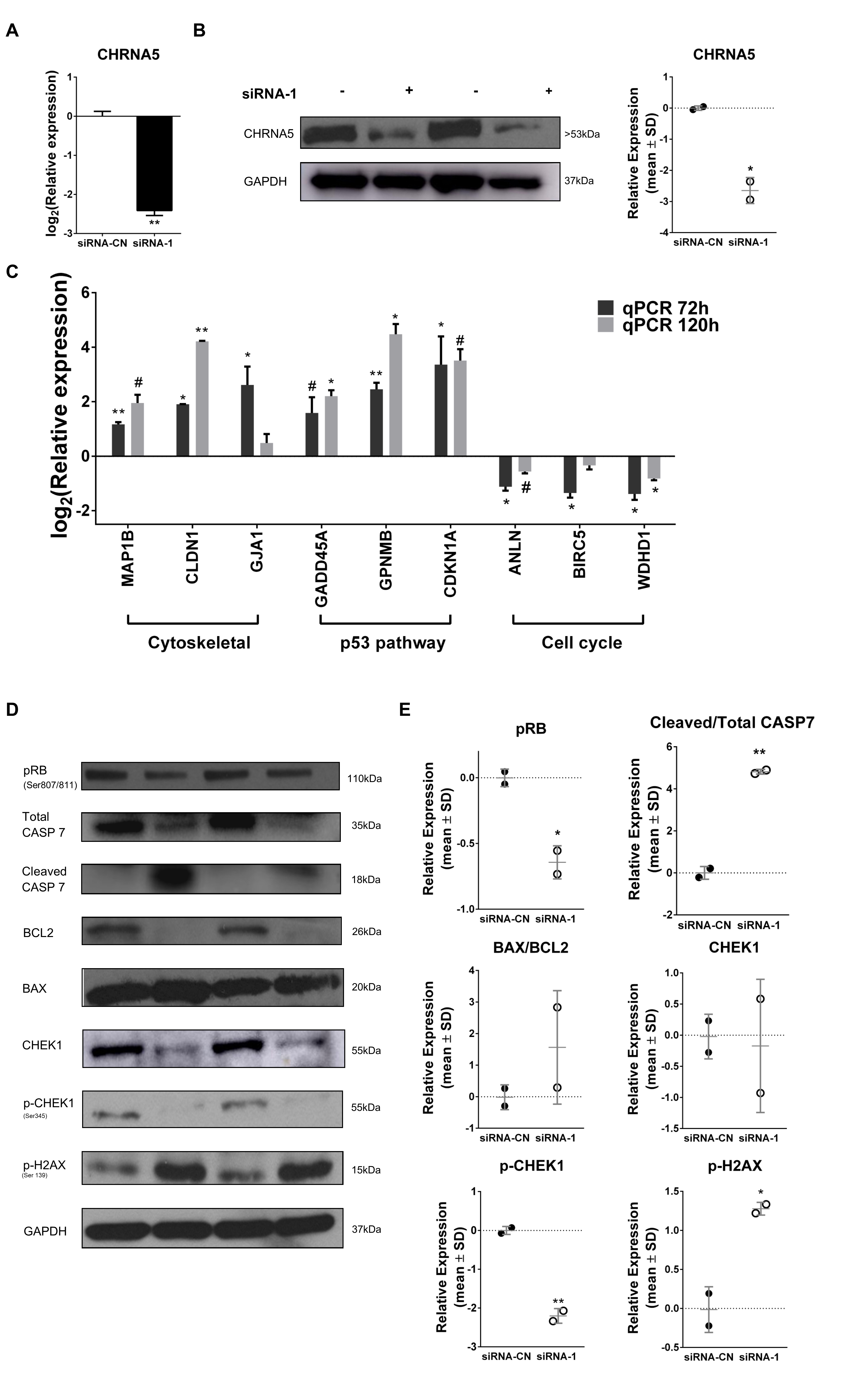

Supplement: S1 Fig — A. RT-qPCR analysis of CHRNA5 depletion for CHRNA5_v1 isoform. B. Western Blotting of siRNA-1 and siRNA-CN (10nM) treated MCF7 cells. C. RT-qPCR analysis of selected genes upon 10nM siRNA-1 treatment for 120h in comparison with 72h values from Fig 4A in MCF7 cells (n = 1 for siRNA-CN and n = 2 for siRNA-1). Expression of SDHA gene is used as reference. D. Western blot analysis of pRB, total CASP7, cleaved CASP7, BCL2, BAX, total CHEK1, pCHEK1, pH2AX proteins from siRNA-1 and siRNA-CN treated groups (n = 2 per group). E. Densitometry analysis and statistical comparisons. Student’s t-test was applied. (*: p < 0.05, **: p < 0.01, #: p < 0.001). (TIF) [file pone.0208982.s006.tif]

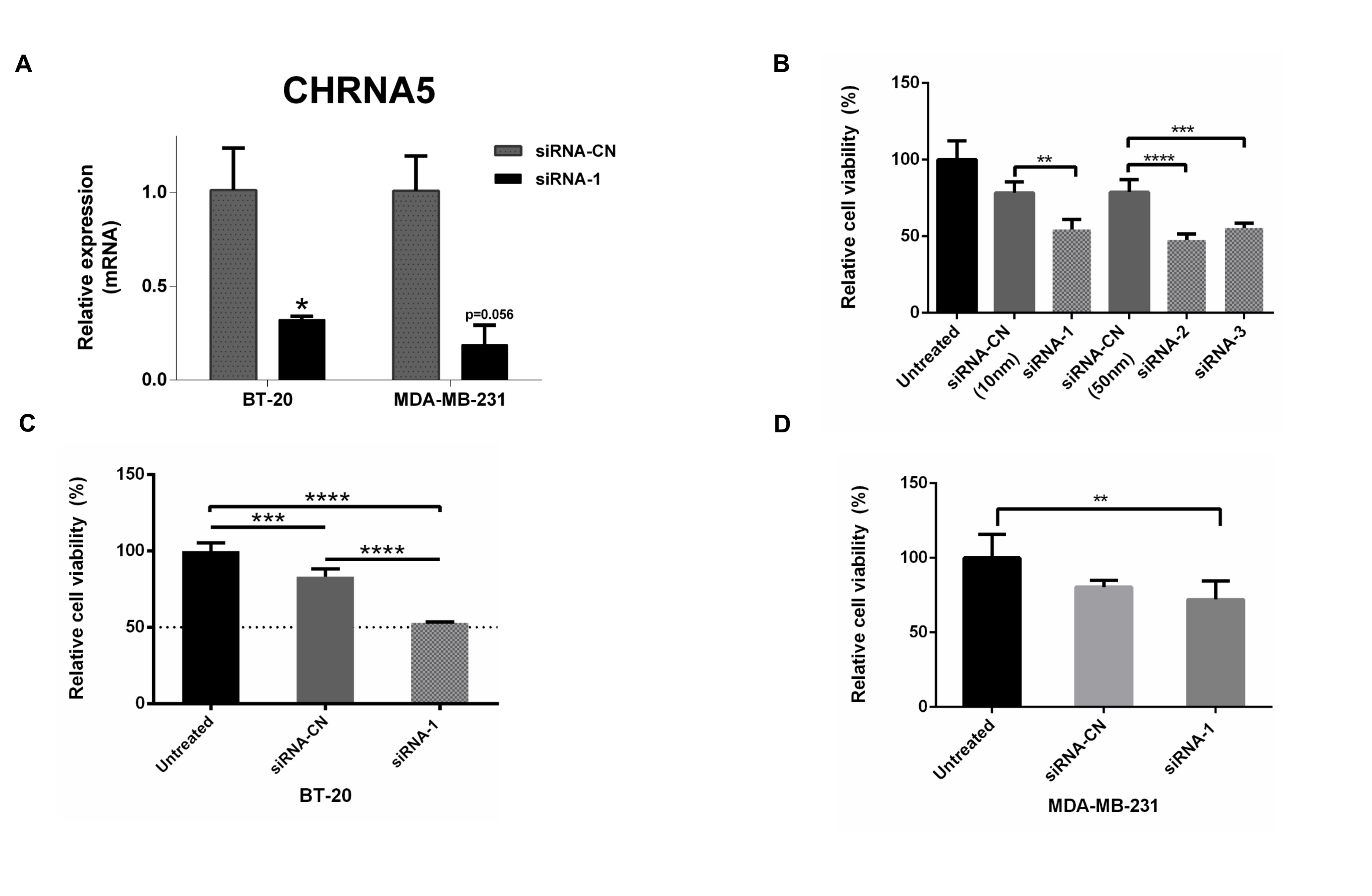

Supplement: S2 Fig — A. CHRNA5 levels in siRNA-1 treated BT20 and MDA-MB-231 cell line (n = 2 per group). B. Relative cell viability of MCF7 cells upon siRNA-1-3 exposure (n = 3 per group). C-D. Relative cell viability of BT20 (C) MDA-MB-231 upon siRNA-1 exposure (D) (n = 3 per group). (*: p < 0.05, **: p < 0.01, ***: p < 0.001, ****: p < 0.0001). (TIF) [file pone.0208982.s007.tif]

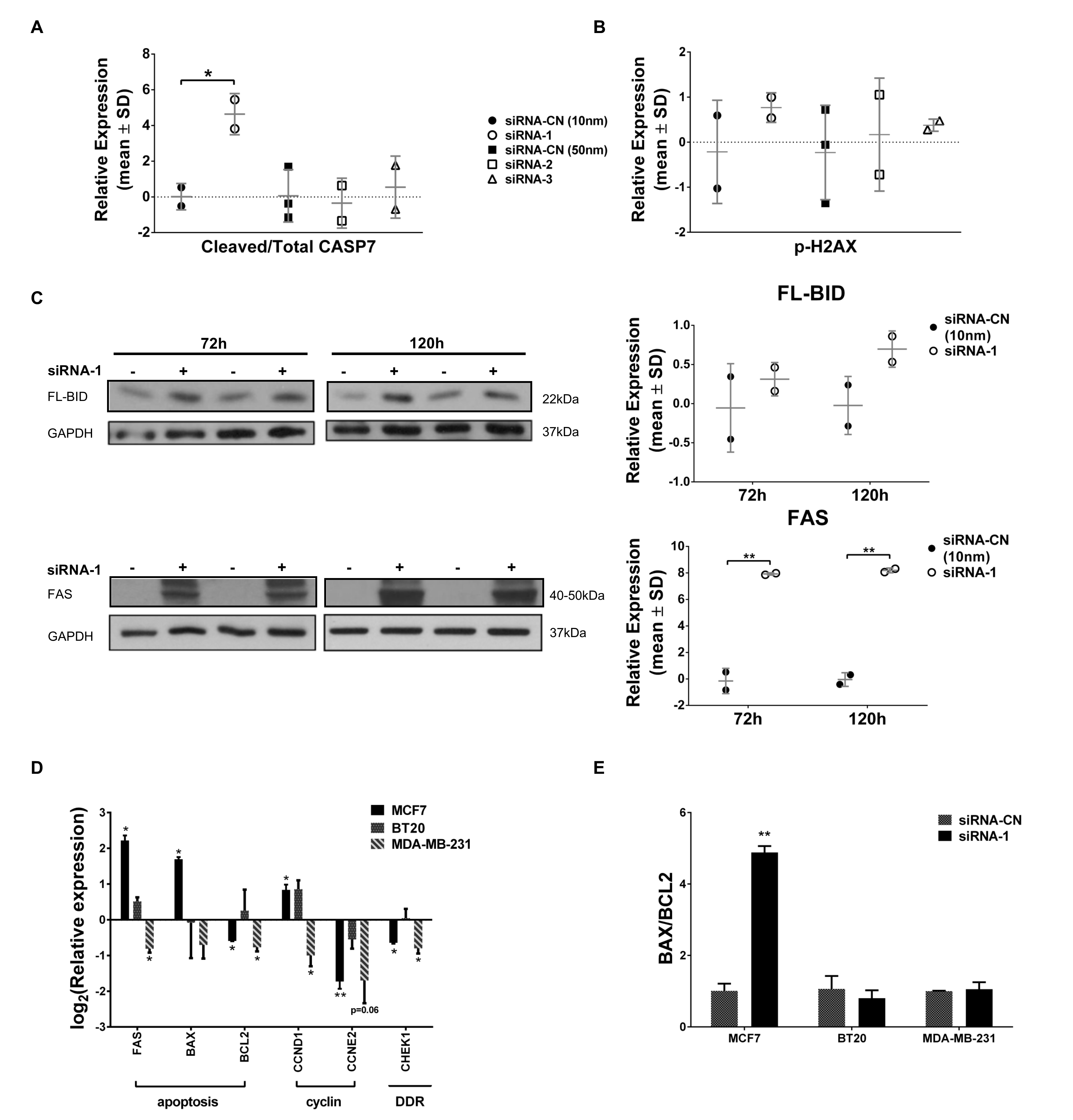

Supplement: S3 Fig — A-B. One-Way ANOVA of densitometry measurements of cleaved CASP7/total CASP7 ratio (A) and pH2AX (B) in MCF7 cells. siRNA-CN (10nM) and siRNA-CN (50nM) were used as control groups for siRNA-1, and siRNA-2-3, respectively. (n = 2 per group for siRNA-CN (10nM) and siRNA-1; n = 3 per group for siRNA-CN (50nM) and siRNA-2 and -3). C. FAS and BID protein levels upon siRNA-1 treatment for 72h (left) and 120h (right) in MCF7 cells and densitometry analysis with student’s t-test. D. RT-qPCR analysis of selected genes after 10nM siRNA-1 treatment for 72h in MDA-MB-231 and BT-20 cells in comparison with results from MCF7 shown Fig 6I. E. BAX/BCL2 ratio in BT-20 and MDA-MB-231 in comparison with MCF7 cells (data from Fig 6J), after siRNA-1 exposure (*: p < 0.05, **: p < 0.01). (TIF) [file pone.0208982.s008.tif]

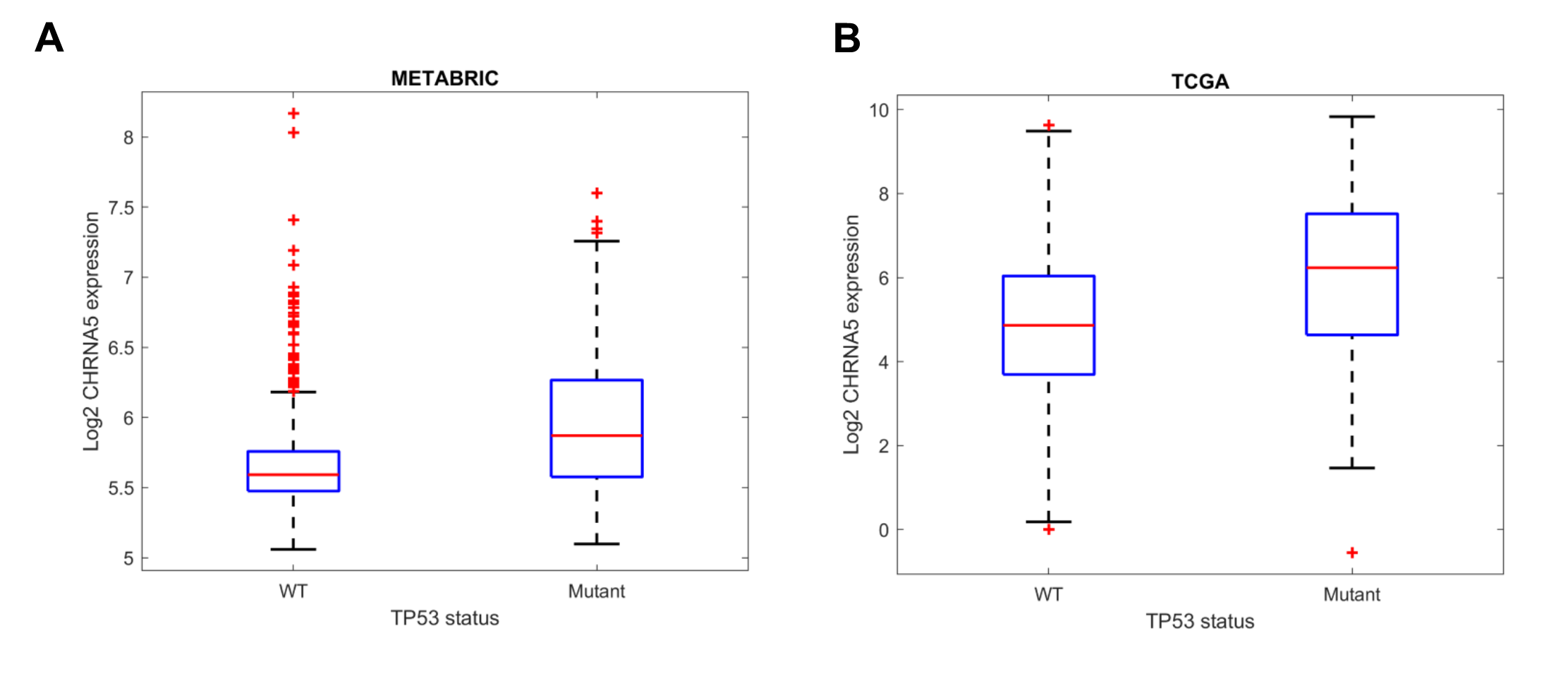

Supplement: S4 Fig — A-B METABRIC (A) and TCGA(B) datasets for TP53 mutant and wild type patients. (TIF) [file pone.0208982.s009.tif]

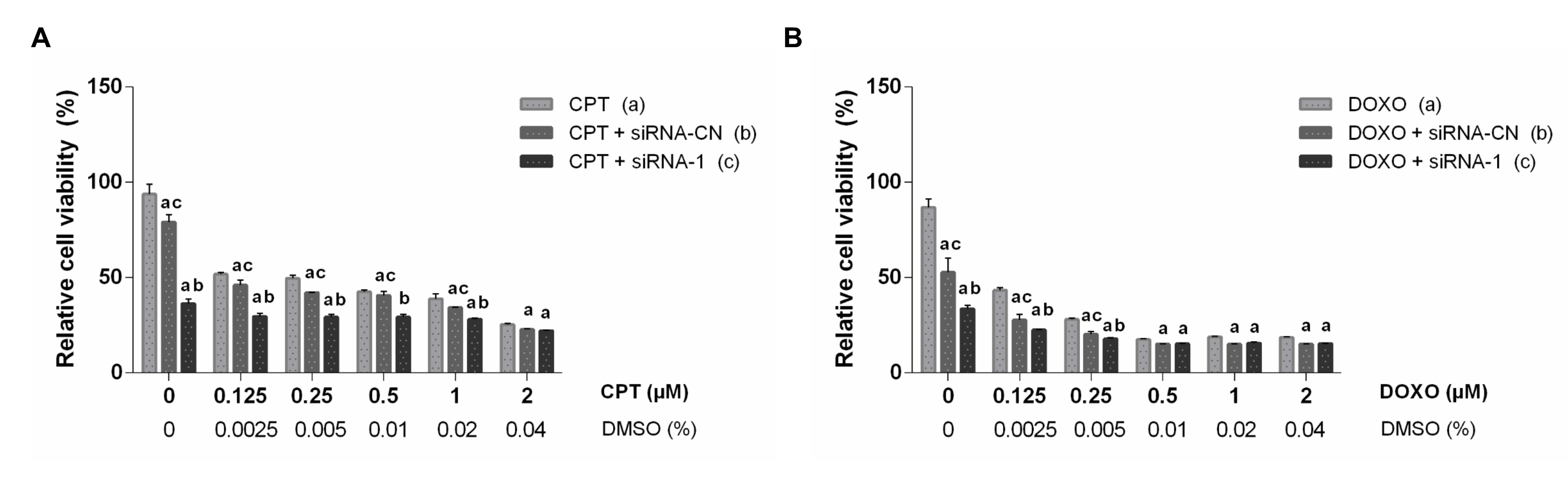

Supplement: S5 Fig — A-B. Relative cell viability of 72h exposure of CPT (0-2uM) (A) or DOXO (0-2uM) (B) and 10nM siRNA-1 treated MCF7 cells, or in combination with the corresponding siRNA-CN controls. Treatments having the same drug and DMSO concentrations were shown on the x-axis as groups; Labels: drug alone (a), drug+siRNA-CN (b), and drug+siRNA-1(c). Letters on top of the siRNA-CN (b) or siRNA-1 exposed (c) treatments are labels indicating the treatment identity (a, b, or c, as defined above) significantly different based on Tukey HSD corrected One-Way ANOVA results (n = 3 per group; p adj. <0.05). (TIF) [file pone.0208982.s010.tif]

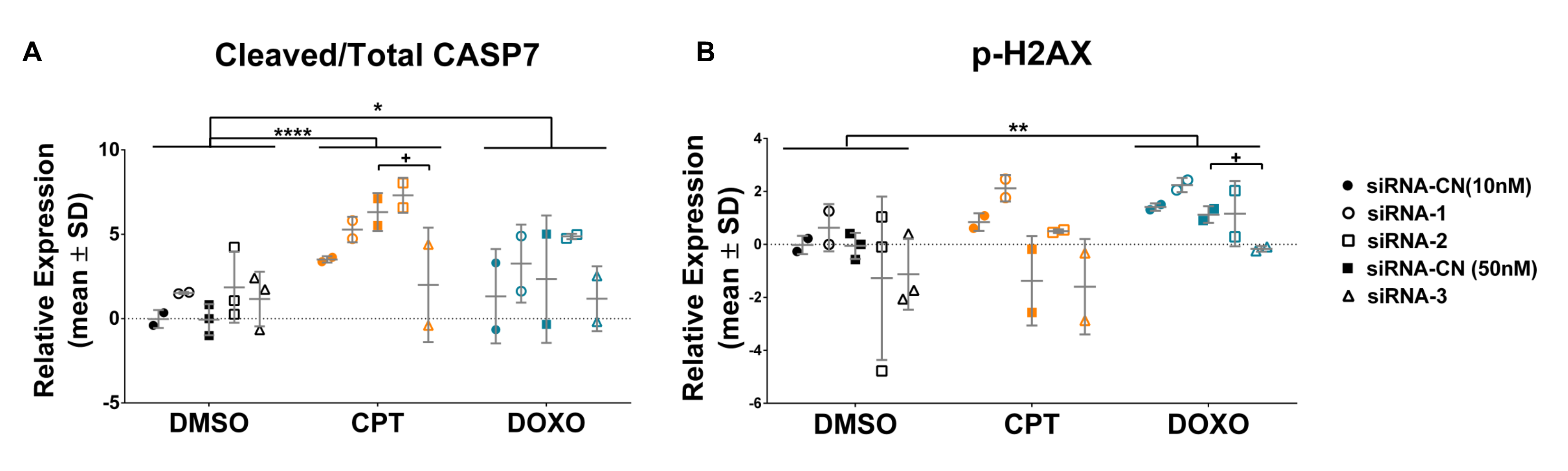

Supplement: S6 Fig — A-B. Two-Way ANOVA of densitometry measurements of cleaved CASP7/total CASP7 (A) and pH2AX (B) in DMSO, CPT, and DOXO groups. (TIF) [file pone.0208982.s011.tif]
